# PuMYB21/PuMYB54 coordinate to activate *PuPLDβ1* transcription during peel browning of cold-stored “Nanguo” pears

**DOI:** 10.1038/s41438-020-00356-3

**Published:** 2020-09-01

**Authors:** Hua-Jun Sun, Man-Li Luo, Xin Zhou, Qian Zhou, Yang-Yang Sun, Wan-Ying Ge, Miao-Miao Yao, Shu-Juan Ji

**Affiliations:** Department of Food Science, Shenyang Agricultural University, 110866 Shenyang, People’s Republic of China

**Keywords:** Plant molecular biology, Transcription

## Abstract

Refrigeration is commonly used to extend the storage life of “Nanguo” pears, but fruit in long-term refrigeration is prone to peel browning, which is related to membrane lipid degradation. To determine the mechanism of membrane lipid degradation, we identified two R2R3-MYB transcription factors (TFs), PuMYB21 and PuMYB54, from “Nanguo” pears, which were notably expressed in response to cold stress and during the peel-browning process. The results from yeast one-hybrid, electrophoretic mobility shift, and transient expression assays indicated that both PuMYB21 and PuMYB54 directly bind to the promoter of *PuPLDβ1* (a key enzyme catalyzing the hydrolysis of membrane phospholipids) and activate its expression, which probably enhances the degradation of membrane phospholipids and eventually results in peel browning. Moreover, the overexpression of PuMYB21 and PuMYB54 can greatly activate the transcription of endogenous *PuPLDβ1* in both “Nanguo” pear fruits and calli, and their silencing can inhibit its transcription. Furthermore, yeast two-hybrid, bimolecular fluorescence complementation, and pull-down assays verified that PuMYB21 interacts with PuMYB54 to enhance the expression of *PuPLDβ1*. In summary, we demonstrate that PuMYB21 and PuMYB54 may have roles in membrane lipid metabolism by directly binding to the downstream structural gene *PuPLDβ1* during the low temperature-induced peel browning of “Nanguo” pears.

## Introduction

Low temperature storage is routinely used to extend the postharvest life of fruit; however, various chilling injury (CI) symptoms commonly occur in fruits subjected to inappropriately low temperatures or long-term cold storage^1^. “Nanguo” pears (*Pyrus ussuriensis* Maxim.) are specialty fruits in the Liaoning Province of China and are mainly produced in the Anshan region. “Nanguo” pears are typically harvested in the middle of September^2^. Although the fruits can be stored for only ~20 d at room temperature^3^, cold storage technology can extended their shelf life to several months^4,5^. However, peel browning is one of the most typical CI symptoms in long-term low temperature-stored “Nanguo” pears, which seriously affects the quality and commercial value of the fruit^4^. Thus, the exploration into the mechanism of peel browning in refrigerated “Nanguo” pears is of great significance.

The cytomembrane, a critical structure in cells, is important in providing a stable internal environment for the life activities of cells, nutrient exchange, and cell recognition^6^. Furthermore, the cytomembrane is critical for cellular and subcellular compartmentalization, preventing contact between substrates and enzymes in the process of enzymatic browning^7–9^. In addition, membrane lipids have significant roles in the adaptation of plants to cold stress^10,11^. Phospholipids are fundamental molecules for establishing biological membranes and are highly conserved in bacteria, mammals, and seed plants^12^. The degradation of membrane lipids can destroy the structural integrity of the cytomembrane and lead to the disruption of cellular compartmentalization, resulting in enzymatic browning caused by the contact of phenolics with polyphenol oxidase^4,6,9,10,13^. It has been reported that phospholipase D (PLD), lipoxygenase (LOX), and lipase contribute to the degradation of membrane lipids^9^. Among these enzymes, PLD catalyzes the hydrolysis of phospholipids, such as phosphatidylcholine (PC) and phosphatidylethanolamine (PE), producing a free head group and phosphatidic acid (PA), which can cause membrane fusion and cell death due to the generation of hydroperoxides and free radicals^14^. In addition, a number of studies have revealed that PLD has a crucial role in regulating the composition of membrane lipids during cold stress^15^. Unsaturated fatty acids (USFAs) maintain membrane integrity upon chilling by reducing the melting temperature and improving membrane fluidity^16^. An increase in polyunsaturated lipids was detected in cold-acclimated *Arabidopsis thaliana*^14^. Our previous research showed that the browning of “Nanguo” pears is related to cellular membrane system damage, such as the degradation of cellular membrane phospholipids and the peroxidation of USFAs^3,17,18^. Under cold stress, the lipid composition changes to become conducive to cytomembrane stabilization. In low temperature-stored bell peppers, increases in the percentage of PA and PLD activity were detected, which indicated that the PLD pathway had been activated and the membrane lipids had undergone remodeling during cold stress^6^. There are many PLD family members in plants, including PLDα, PLDβ, PLDδ, PLDε, and PLDζ^19,20^. *AtPLDα1* has been reported to hydrolyze structural phospholipids into PA, and the suppression of *AtPLDα1*-induced freezing tolerance in *A. thaliana* under cold stress^19^. However, *OsPLDα1*, which is 79% identical to *AtPLDα1* at the amino acid level, has opposite roles as those of *AtPLDα1* in regulating cold stress responses. Genetic studies have shown that reducing the transcription of *OsPLDα1* increased the chilling stress sensitivity of rice plants^21^. In addition, the transcription level of bell pepper *CaPLDα4* increased in response to cold stress^6^. Recent research has shown that *CaPLDα4* is regulated by the transcription factor (TF) CaNAC1, which binds to the promoter of *CaPLDα4* and activates its transcription^20^. The genetic knockout of *AtPLDδ* rendered *A. thaliana* plants more sensitive to freezing, whereas the overexpression of *AtPLDδ* increased its freezing tolerance, and lipid profiling revealed that the overexpression of *AtPLDδ* increased the production of PA species^21^. It was reported that the knockdown of *AtPLDβ* enhanced the cold tolerance of *A. thaliana*^22^. However, the transcriptional regulation of phospholipid degradation-related genes by upstream regulators was given little acknowledgment and is a scientific aspect that needs further exploration.

TFs are proteins that have the capacity to regulate gene transcription by binding the specific nucleotide upstream sequence of the 5′-end of a gene upon sensing abiotic stressors, including low temperature^23^. Commonly, the nuclear localization signal, transcription-activation domain, DNA-binding domain, and oligomerization sites are the four domains that constitute TFs^24^. In addition, TFs affect many multigene family members, greatly increasing the complexity of transcriptional regulation^24^. Higher plants contain a wide range of TFs, among which MYB TFs constitute one of the largest TF families, accounting for ~9% of all plant TFs and participating in the regulation of many aspects of plant growth, development, metabolism, and stress responses^25^. Most MYB family members share a highly conserved MYB domain in the N terminus^26^. The DNA-binding domain in MYB TFs is typically composed of 50–53 amino acid residues, which each form a domain consisting of three α-helices^27^. According to an analysis of the three-dimensional structure of the MYB domain, the second and third helices of each repeat have three regularly spaced tryptophan (W) residues forming a helix-turn-helix (HTH) structure with a hydrophobic core, whereas the third helix directly interacts with the major groove of the target DNA^28,29^. Based on the number of repeats in MYB domains, MYB superfamily members are classified into four primary subfamilies: MYB-related proteins (1R-MYB, one repeat), R2R3-MYB proteins (2R-MYB, two repeats), R1R2R3-MYB proteins (3R-MYB, three repeats), and 4R-like MYB protein (4R-MYB, four repeats)^30^. Among these types of MYB TFs, R2R3-MYB is the major subfamily^30^. R2R3-MYB TFs are characterized by various functions in regulating several aspects of biological processes, and many MYB TFs are involved in regulating responses to abiotic stresses, including cold stress^31^. In apple, the transcription of MdMYB23 is induced by cold stress, and the overexpression of MdMYB23 increases the cold tolerance of transgenic apple calli and *A. thaliana* by activating the expression of C-repeat binding factors (CBFs), including MdCBF1 and MdCBF2^32^. In addition, MdMYB88 and MdMYB124 positively regulate cold hardiness and cold-responsive gene expression under cold stress through CBF-dependent and CBF-independent pathways^33^. Peel browning is caused by cold stress; however, the involvement of MYB TFs in peel browning has been largely unexplored.

In this work, we report the functions of PuMYB21 and PuMYB54 in “Nanguo” pears, which are both induced by cold stress. Biochemical experiments showed that both PuMYB21 and PuMYB54 bind directly to the *PuPLDβ1* promoter in vivo and in vitro and then activate the expression of *PuPLDβ1*. Therefore, we propose a model of increasing abiotic tolerance based on a membrane lipid metabolism feedback loop in response to cold stress. This study provides insight into the regulatory mechanisms of MYB TFs under cold stress in plants.

## Results

### Peel browning

**Fig. 1:**
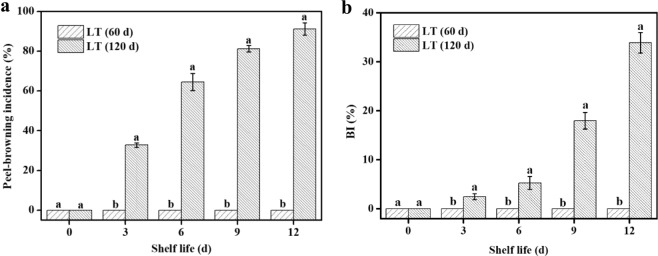
Changes in peel browning. **a** Peel-browning incidence. **b** Peel-browning index (BI) of “Nanguo” pears during the refrigeration and subsequent shelf life periods. LT (60 d) and LT (120 d) indicate pears maintained at low temperature (0 ± 0.5 °C) for 60 and 120 d, respectively. The values represent the mean and corresponding SD (*n* = 3). Different letters indicate a 5% level of significance as determined by pairwise Student’s *t*-test

To estimate the severity of CI, we calculated the BI and peel-browning incidence of cold-stored “Nanguo” pears during the refrigeration and subsequent shelf life periods. As shown in Fig. 1a, b, no peel browning was observed in the fruits stored for 60 d during the entire shelf life period, whereas the fruit stored for 120 d showed browning on the 3rd day of the shelf life period. The browning incidence and BI index increased greatly with the extension of shelf life time.

### Changes in membrane phospholipids

The fruit refrigerated for 120 d showed obvious browning on the 6th day of the shelf life period, whereas no significant differences in ripeness were observed between the fruits refrigerated for 60 and those refrigerated for 120 d. We detected five key phospholipids, namely, phosphatidic acid (PA), phosphatidylcholine (PC), phosphatidylethanolamine (PE), phosphatidylserine (PS), and phosphatidylglycerol (PG), in the fruit stored for different periods (Table 1). The total phospholipid content level in the pears refrigerated for 120 d was much lower than it was in the short-term-refrigerated pears, which indicated that low temperature stress promotes the degradation of phospholipids in the peel tissues and may be closely associated with the peel browning of the refrigerated “Nanguo” pears. Furthermore, the distribution of phospholipids in the samples refrigerated for 60 and 120 d was different. The content levels of PA and PS in the samples refrigerated for 120 d were lower than they were in the samples refrigerated for 60 d; however, their percentages were significantly higher in pears refrigerated for 120 d. In addition, the content levels and percentages of PG, PC, and PE were reduced after long-term refrigeration.

**Table 1:** Phospholipid composition and proportion of “Nanguo” pears on shelf life 6 d after being in cold storage for 60 and 120 d

Lipid class	Lipid/dry weight (nmol mg^−1^)		Relative proportion to total phospholipids (%)	
	LT (60 d)	LT (120 d)	LT (60 d)	LT (120 d)
PA	1.55 ± 0.02^a^	1.41 ± 0.02^b^	26.40 ± 3.27^b^	36.51 ± 2.58^a^
PG	0.91 ± 0.01^a^	0.58 ± 0.02^b^	15.47 ± 2.15^a^	15.09 ± 2.17^a^
PS	0.11 ± 0.01^a^	0.10 ± 0.01^a^	1.95 ± 0.56^b^	2.54 ± 0.89^a^
PC	1.97 ± 0.03^a^	1.02 ± 0.01^b^	33.56 ± 4.33^a^	26.35 ± 1.96^b^
PE	1.33 ± 0.02^a^	0.75 ± 0.02^b^	22.65 ± 4.12^a^	19.48 ± 2.25^b^
Total lipids	5.88 ± 0.33^a^	3.86 ± 0.42^b^	100^a^	100^a^

The relative proportion of phospholipids is the percentage of the dry weight of each polar lipid with respect to the total dry weight of all the lipids. LT (60 d) and LT (120 d) indicate pears kept at low temperature (0 ± 0.5 °C) for 60 and 120 d, respectively. The values represent the mean and corresponding SD (*n* = 3). Different letters a and b indicate a 5% level of significance between different cold storage times as determined by pairwise Student’s *t*-test.

### Changes in the expression of PLD family members in “Nanguo” pears stored for different times

**Fig. 2:**
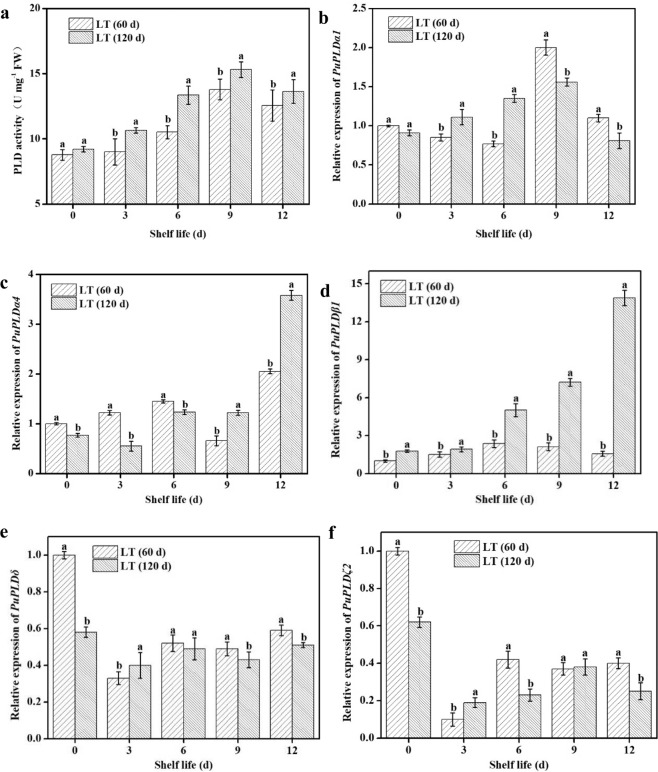
Activity and expression analysis of PuPLDs. **a** Activity of PLD. Expression patterns of **b** *PuPLDα1*, **c** *PuPLDα4*, **d** *PuPLDβ1*, **e** *PuPLDδ*, and **f** *PuPLDζ2* in “Nanguo” pears during the refrigeration and subsequent shelf life periods. β-Actin was the reference gene. LT (60 d) and LT (120 d) indicate pears maintained at low temperature (0 ± 0.5 °C) for 60 and 120 d, respectively. The values indicate the mean and corresponding SD (*n* = 3). Different letters indicate a 5% level of significance as determined by pairwise Student’s *t*-test

To analyze the effects of low temperature stress on different PLD family members, this study tested the changes in PLD activity and expression of PLD family members, including *PuPLDα1*, *PuPLDα4*, *PuPLDβ1*, *PuPLDδ*, and *PuPLDζ2*, in “Nanguo” pears stored at room temperature after different periods of refrigeration. As shown in Fig. 2a, the PLD activity level in the samples stored for 120 d was significantly higher than that in the samples stored short-term, except on the first day of shelf life. In terms of the expression of PLD, the performance of different family members was significantly different. *PuPLDδ* and *PuPLDζ2* demonstrated similar trends (Fig. 2e, f). On the day the samples were removed from cold storage, the expression level in the short-term stored pears was significantly higher than that in the long-term-stored samples. However, when the pears were transferred to shelf life, the expression level decreased rapidly and vacillated in the long-term-refrigerated samples. The differences in the expression in *PuPLDα1* in the pears during different storage periods were mainly reflected in the middle and late stages of shelf life, with the expression of *PuPLDα1* in the middle stage of the shelf life after long-term refrigeration being higher than that after short-term storage, and the trend in the later shelf life period was exactly the opposite (Fig. 2b). The expression of *PuPLDα4* in the early and middle shelf life period was higher in the long-term-refrigerated fruit than it was in the fruit stored for 60 d, and int the later shelf life period, the expression of *PuPLDα4* was significantly higher in the long-term-stored fruit than it was in the short-term-stored fruit (Fig. 2c). In contrast to other PLD family members, the expression of *PuPLDβ1* in the short-term-stored pears remained low throughout the shelf life period, whereas the expression of *PuPLDβ1* in the pears after long-term storage gradually increased throughout the shelf life period, especially in the middle and late stages (Fig. 2d).

### Isolation and motif analysis of the *PuPLDβ1* promoter

To identify the *cis*-elements in the promoter sequences, a 700 bp region upstream of the translation start site in the *PuPLDβ1* gene was isolated based on the homologous gene of the Chinese white pear and identified using PlantCARE. In addition to the core *cis*-acting elements, such as a CAAT and TATA box motif, an MYB-binding site (MBS) with a core sequence (CAACTG), WRKY-binding site W-Box, and bHLH-binding site G-Box were identified in the *PuPLDβ1* promoter, suggesting the possible involvement of MYB, WRKY, and bHLH TFs in regulating *PuPLDβ1*. Binding sites for AREB/ABF TFs were also identified (Table 2).

**Table 2:** Main regulatory motifs in the *PuPLDβ1* promoter

Factor or site name	Signal sequence	Probable function
ABRE	CACGTG; ACGTG; TACGTG; CACGTA	Abscisic acid-responsive element
as-1	TGACG	Transcription factor-binding site
CAAT-box	CCAAT	Common *cis*-acting element in the promoter and enhancer regions
ERE	ATTTTAAA	ERF (ethylene response factor) binding site
G-box	CACGTT	Involved in light responsiveness
MBS	CAACTG	MYB-binding site
TATA-box	TATA; TATAAA; TATAA	Core promoter element approximately −30 from the transcription start site
TCA-element	CCATCTTTTT	Salicylic acid-responsive element
TGACG-motif	TGACG	MeJA-responsive element
W-box	TTGACC	WRKY-binding site
WRE3	CCACCT	WRE transcription factor-binding site
WUN-motif	AAATTTCTT	WUN transcription factor-binding site

### Interaction of PuMYB21 and PuMYB54 with the PuPLDβ1 promoter

**Fig. 3:**
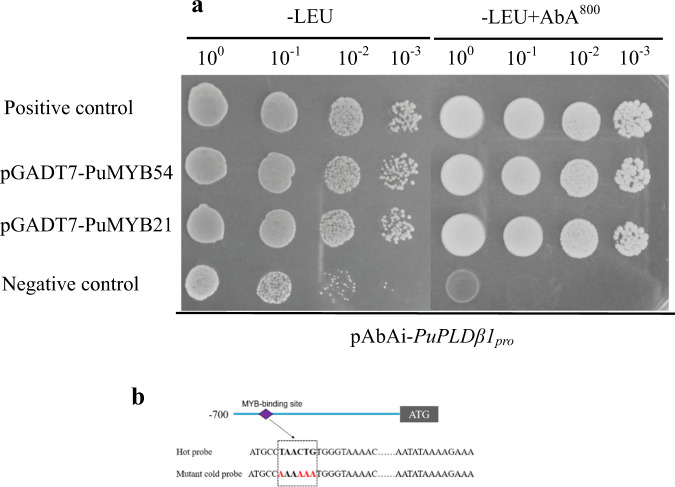
PuMYB21 and PuMYB54 bound to the *PuPLDβ1* promoter. **a** The growth status of transformed yeasts on two types of media. Normal yeast growth on defective medium containing the antibiotic aureobasidin A (−Leu + AbA^800^) indicates the binding of PuMYB21 and PuMYB54 to the promoter of *PuPLDβ1*. **b** Electrophoretic mobility shift assay (EMSA) results showing the interaction between PuMYB21/54 and the labeled probes in the *PuPLDβ1* promoter. Bold letters indicate the MYB-binding site (MBS), which were biotin-labeled (hot probe). Red letters represent mutated MBS (mutant cold probe). The cold probe represents an unlabeled sequence. The symbols + or − represent presence or absence, respectively, and 200× indicates the amount of cold probe

To verify the binding of the candidate transcription factor proteins to the *PuPLDβ1* promoter, based on the transcription factor-binding site on the *PuPLDβ1* promoter and our transcriptome results, 17 transcription factors from 3 families (PuMYB4, PuMYB6, PuMYB21, PuMYB24, PuMYB44, PuMYB54, PuMYB66, PuMYB84, PuMYB86, PuMYB91, PuMYB306, PuMYB1R1, PubHLH13, PubHLH15, PubHLH21, PubHLH66, and PuWRKY20) were selected for use in the yeast one-hybrid (Y1H) assay. Only the yeast strains with PuMYB21 and PuMYB54 proteins were able to grow on the cultivation medium containing AbA (Fig. 3a); the other TFs did not interact with *PuPLDβ1* (Supplementary Fig. S1).

To investigate the specific binding sites, the full-length PuMYB21 and PuMYB54 proteins were purified, and EMSA was conducted with a biotin-labeled *PuPLDβ1* promoter fragment containing the MYB-binding motif used as the labeled probe. PuMYB21 and PuMYB54 bound to the *PuPLDβ1* promoter, and when an unlabeled probe containing mutated nucleotides was added as a competitor, the binding of PuMYB21 and PuMYB54 to the *PuPLDβ1* promoter was not affected (Fig. 3b). This result indicates that PuMYB21 and PuMYB54 can physically bind to the MBS motif in the *PuPLDβ1* promoter.

### PuPLDβ is transcriptionally activated by PuMYB21 and PuMYB54

**Fig. 4:**
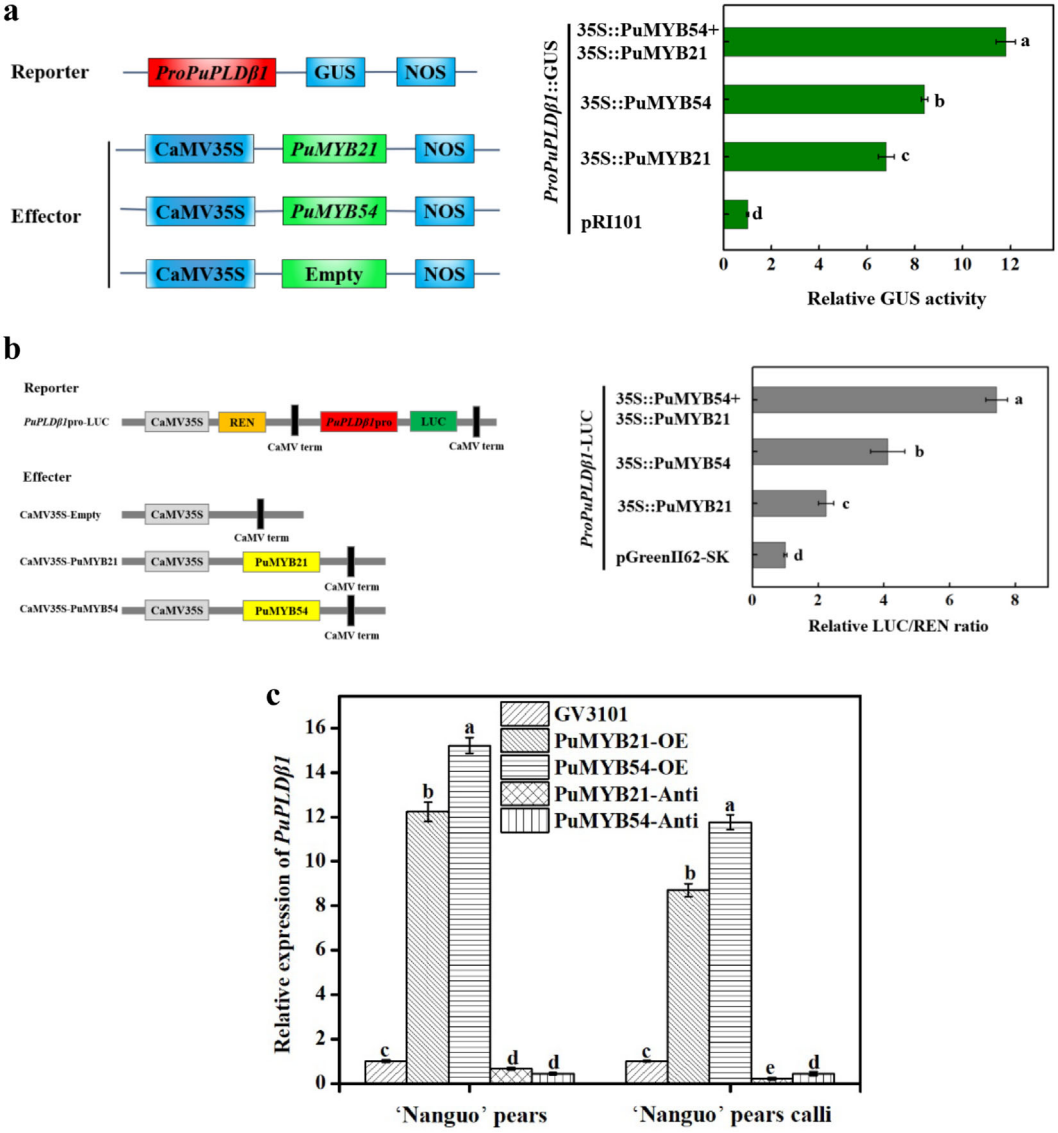
PuMYB21 and PuMYB54 enhanced the activity of the *PuPLDβ1* promoter in transient expression assays with tobacco leaves and “Nanguo” pears and calli. **a** *Agrobacterium tumefaciens* carrying the PuMYB21/54 or *PuPLDβ1* plasmids was infiltrated into tobacco leaves to measure the activity enhancement of the *PuPLDβ1* promoter by PuMYB21 and PuMYB54. The effector and empty pRI101 plasmids that were cotransformed into the tobacco leaves were used as the controls. **b** The PuMYB21 and PuMYB54 effectors with the *PuPLDβ1* promoter reporter were infiltrated into the tobacco leaves. Significantly higher LUC/REN ratios were observed with the PuMYB21 and PuMYB54 effector vectors than with the empty control vector, indicating that PuMYB21 and PuMYB54 enhanced *PuPLDβ1* promoter activity. **c** The relative expression of *PuPLDβ1* in the overexpressed or silenced “Nanguo” pears and calli. GV3101, *A. tumefaciens* strain GV3101; PuMYB21-OE and PuMYB54-OE, overexpressed PuMYB21 and PuMYB54; PuMYB21-Anti and PuMYB21-Anti, inhibited PuMYB21 and PuMYB54. Values are the means ± SD (*n* = 3, *n* represents the number of pears and calli, respectively), and letters represent significance at *P* < 0.05 compared with the control based on one-way analysis of variance (ANOVA) followed by Duncan’s multiple range test

These results demonstrate that PuMYB21 and PuMYB54 can bind to the promoter of *PuPLDβ1*. To further examine whether the transcriptional activity of *PuPLDβ1* was activated or suppressed by PuMYB21 and PuMYB54, a GUS transactivation experiment was performed in wild tobacco (*Nicotiana benthamiana*) leaves. The CDSs of PuMYB21 and PuMYB54 were inserted into a pRI101 vector, and the promoter fragments of *PuPLDβ1* were inserted into a pBI101 vector. A significant increase in GUS activity level was observed, compared with the control, when 35S::PuMYB21+*ProPuPLDβ1*::GUS and 35S::PuMYB54+*ProPuPLDβ1*::GUS were coexpressed (Fig. 4a). Furthermore, we found that there was significantly higher relative GUS activity in the coexpressed 35S::PuMYB21+35S::PuMYB54+*ProPuPLDβ1*::GUS tobacco leaves than in the others. These results suggest that PuMYB21 and PuMYB54 enhance the transcription of *PuPLDβ1* by directly interacting with its promoter.

Transient expression assays with tobacco leaves using a dual-luciferase reporter were also performed. The CDSs of PuMYB21/54 were cloned into pGreen II 62-SK, and the promoter fragment of *PuPLDβ1* was inserted into pGreen II 0800-LUC. The data showed that the interaction of PuMYB21 with *PuPLDβ1* led to a nearly 2.2-fold increase in the relative LUC/REN ratio, and the interaction of PuMYB54 with *PuPLDβ1* led to a nearly fourfold increase in the relative LUC/REN ratio (Fig. 4b). Interestingly, there was a 7.4-fold relative LUC/REN ratio in the tobacco leaves injected with PuMYB54 + PuMYB21 + *PuPLDβ1*. These results indicate that PuMYB21 and PuMYB54 are transcriptional activators that regulate the expression of *PuPLDβ1*. In addition, we speculated that PuMYB21 and PuMYB54 most likely interacted with each other.

To elucidate the positive regulation of PuMYB21 and PuMYB54 in activating *PuPLDβ1*, we monitored the expression of *PuPLDβ1* by real-time PCR analysis in “Nanguo” pear fruit and calli transiently transformed with MYB overexpression or silencing constructs. As shown in Fig. 4c, the expression of *PuPLDβ1* was significantly higher in both the PuMYB21-OE and PuMYB54-OE “Nanguo” pears and calli. In contrast, *PuPLDB1* expression was significantly lower in both PuMYB21-Anti and PuMYB54- Anti “Nanguo” pears and calli. These results suggest that both PuMYB21 and PuMYB54 can positively regulate the transcription of endogenous *PuPLDβ1* in “Nanguo” pears.

### Isolation and sequence analysis of PuMYB21 and PuMYB54

**Fig. 5:**
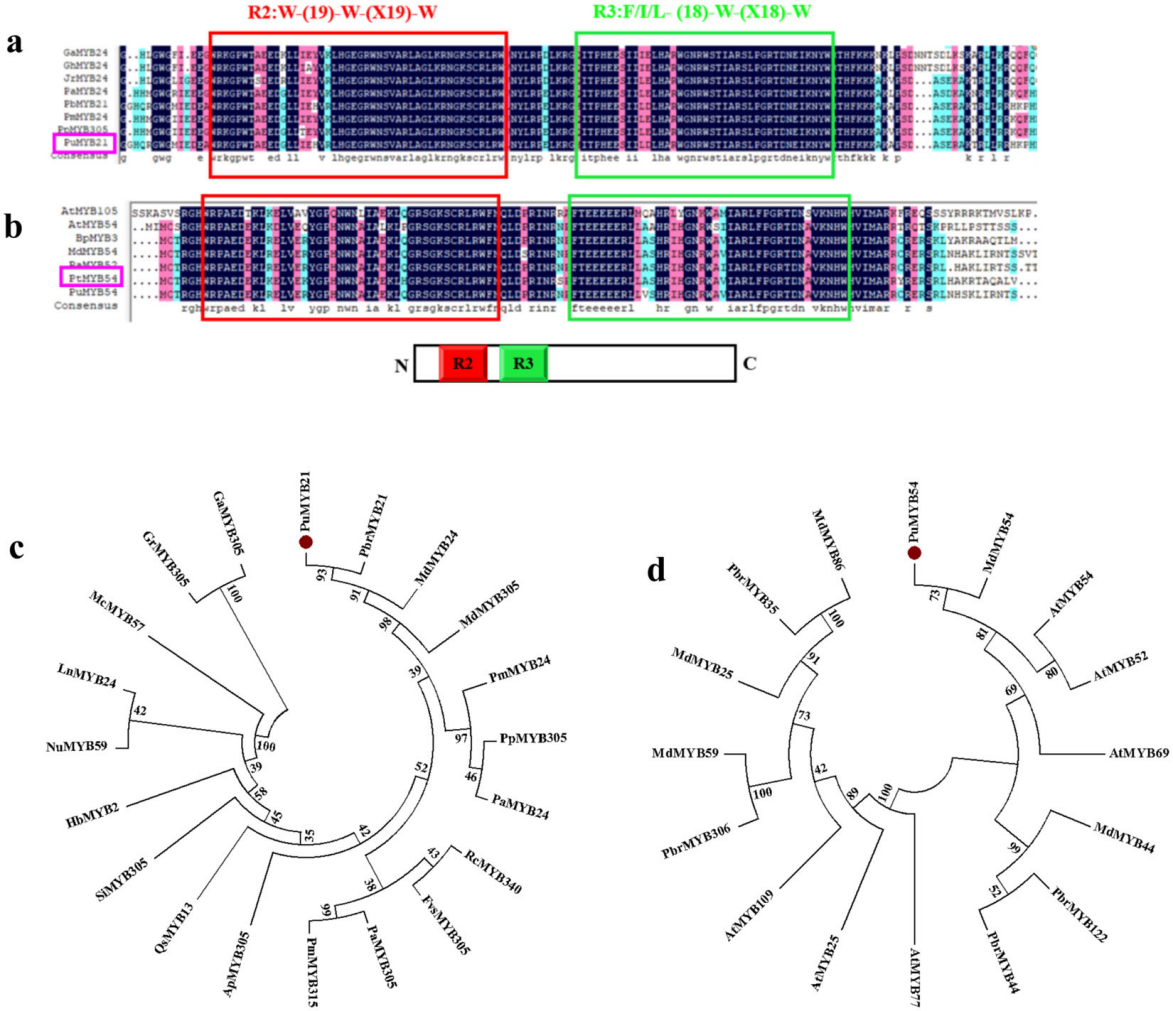
Multiple sequence alignment, phylogenetic analysis, and subcellular localization of PuMYB21 and PuMYB54. **a** Multiple sequence alignment of PuMYB21 and MYBs from other plant species, including *Gossypium arboreum* (GaMYB24, XP_017605542.1), *Gossypium hirsutum* (GhMYB24, AFJ21697.1), *Juglans regia* (JrMYB24, XP_018846808.1), *Prunus avium* (PaMYB24, XP_021806997.1), *Pyrus × bretschneideri* (PbrMYB21, XP_009357956.1), *Prunus persica* (PpMYB305, XP_007221842.1), and *Prunus mume* (PmMYB24, XP_008224020.1). **b** Multiple sequence alignment of PuMYB54 and MYBs from other plant species, including *Arabidopsis thaliana* (AtMYB105, NP_177115.1, and AtMYB54, NP_177484.1), *Betula platyphylla* (BtMYB3, AKN79285.1), *Malus domestica* (MdMYB54, XP_008393477.1), *Prunus avium* (PaMYB52, XP_021802995.1), and *Populus trichocarpa* (PtMYB54, XP_006372770.1). **c** Phylogenetic tree analysis of PuMYB21. The red dot is PuMYB21. **d** Phylogenetic tree analysis of PuMYB54. The red dot is PuMYB54

The increased expression of *PuPLDβ1* in long-term cold-stored fruit suggested that the action of low temperature in accelerating peel browning involves transcriptional regulation. Since the *PuPLDβ1* promoter has MYB-binding motifs, the PuMYB21 and PuMYB54 transcription factors were isolated from “Nanguo” pears. After performing a NCBI BLAST search, we found that PuMYB21 and PuMYB54 were homologous genes of PbrMYB21 (XM_009359681.2) and MdMYB54 (XM_017337313.2), respectively. The PuMYB21 gene is 720 bp and encodes a 239 amino acid polypeptide, and the PuMYB54 gene is 813 bp and encodes a 269 amino acid polypeptide. Multiple sequence alignment indicated that both genes belong to the R2R3-MYB subfamily, as they have two conserved MYB domains: an R2 MYB domain (red box) and an R3 MYB domain (green box) (Fig. 5a, b). A phylogenetic analysis indicated that PuMYB21 and PuMYB54 have high similarity to PbrMYB21 and MdMYB54, respectively (Fig. 5c, d).

### Expression of PuMYB21 and PuMYB54 in “Nanguo” pears during the shelf life period after refrigeration

**Fig. 6:**
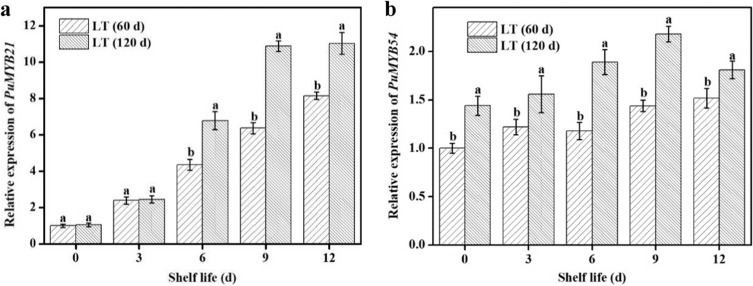
Expression analysis of *PuMYB21* and *PuMYB54*. **a** Expression patterns of *PuMYB21*. **b** Expression patterns of *PuMYB54* in “Nanguo” pears during the refrigeration and subsequent shelf life periods. β-Actin was the reference gene. LT (60 d) and LT (120 d) indicate pears maintained at low temperature (0 ± 0.5 °C) for 60 and 120 d, respectively. The values represent the mean and corresponding SD (*n* = 3). Different letters indicate a 5% level of significance as determined by pairwise Student’s *t*-test

The relative expression levels of the candidate genes *PuMYB21* and *PuMYB54* were tested during the refrigeration and subsequent shelf life periods. The expression of *PuMYB21* in pears during different refrigeration periods increased throughout the shelf life time, whereas the expression in the samples under long-term refrigerated storage were much higher than those under short-term storage during the middle and late periods (Fig. 6a). The expression of *PuMYB54* in the pears refrigerated for 60 d gradually increased throughout the shelf life time. In contrast, the increase range and expression level of *PuMYB54* in pears refrigerated for 120 d were significantly higher than in the short-term-stored pears (Fig. 6b). The reverse-transcription quantitative PCR (RT-qPCR) results showed that the transcription levels of *PuMYB21* and *PuMYB54* were upregulated in response to cold stress and had an expression pattern similar to that of *PuPLDβ1*. From our previous RNA-seq study, the expression of other detected MYBs following long-term cold treatment is shown in Supplementary Table S1.

### The subcellular localization of PuMYB21 and PuMYB54

**Fig. 7:**
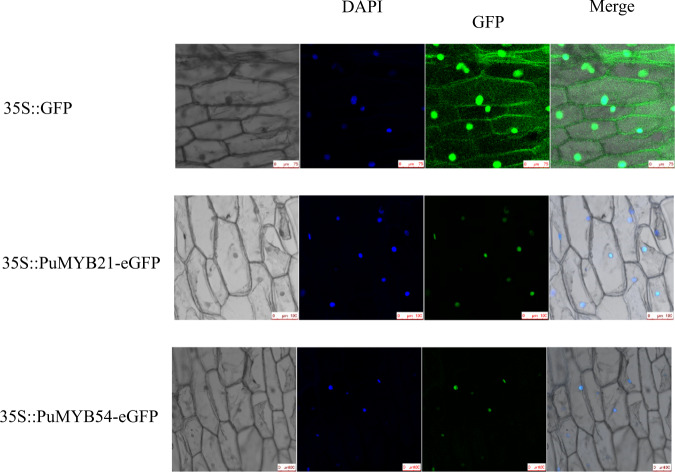
Subcellular localization of the 35S::PuMYB21-eGFP and 35S::PuMYB54-eGFP fusion constructs in onion epidermal cells. 35S::eGFP was used as a control. DAPI is a nuclear dye

To confirm the subcellular localization of PuMYB21 and PuMYB54 in vivo, the CDS regions (without the stop codon) were fused to the eGFP reporter gene in the pRI101 vector. The constructs 35S::PuMYB21-eGFP and 35S::PuMYB54-eGFP were used to infect onion epidermal cells using the *Agrobacterium*-mediated transformation method with 35S::eGFP used as the negative control. The results showed that fluorescence signals from the 35S::PuMYB21-eGFP and 35S::PuMYB54-eGFP fusion proteins were exclusively localized to the nucleus, whereas 35S::eGFP was ubiquitously distributed throughout the cells (Fig. 7).

### PuMYB21 interacts with PuMYB54

**Fig. 8:**
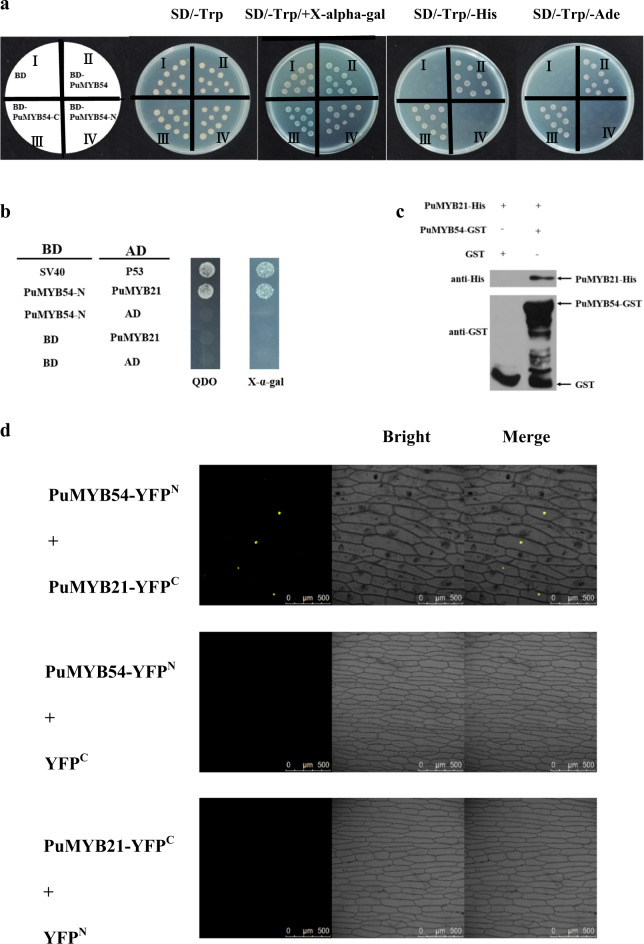
Interaction between PuMYB21 and PuMYB54. **a** The self-activating activity of PuMYB54. I pGBKT7 empty vector. II pGBKT7-PuMYB54 in Y2H. III pGBKT7-PuMYB54C in Y2H. IV pGBKT7-PuMYB54N in Y2H. **b** The N terminus of PuMYB54 interacted with PuMYB21. Y2H Yeast strain containing the bait plasmid PuMYB54N was transformed into PuMYB21. QDO is medium without Leu, Trp, His, or Ade. X-α-gal is QDO with X-α-gal. SV40 and P53 served as the positive control, and the others were negative controls. c A pull-down assay verified the interaction between PuMYB21 and PuMYB54. *Escherichia coli*-expressing GST or PuMYB54-GST fusion proteins were incubated with cobalt chelate affinity resin containing the immobilized histidine-tagged MdBT2 protein. The protein mixtures were purified using a HIS purification kit. **d** Bimolecular fluorescence complementation assays showing the interaction between PuMYB21 and PuMYB54 in the nuclei of onion epidermal cells

To verify that PuMYB21 can interact with PuMYB54, a Y2H assay was performed. We constructed the pGBKT7-PuMYB54 bait vector and determined its self-activating activity. The results showed that PuMYB54 had self-activating activity; therefore, PuMYB54 was separated into two domains and inserted into a pGBKT7 vector. As shown in Fig. 8a, PuMYB54C also showed self-activating activity, whereas PuMYB54N did not exhibit self-activating activity. Therefore, PuMYB54N was used to identify the interaction between PuMYB54 and PuMYB21. Yeast cells transformed with pGBKT7-PuMYB54N + pGADT7-PuMYB21 grew well and turned blue in SD/-Trp/-Leu/-His/-Ade/+X-alpha-gal, indicating that PuMYB21 interacted with PuMYB54 in yeast cells. Moreover, the N-terminal regions of PuMYB54 interacted with PuMYB21 (Fig. 8b). Then, the recombined poly-histidine-tagged PuMYB21 (PuMYB21-His) and recombinant glutathione *S*-transferase-tagged PuMYB54 (PuMYB54-GST) fusion proteins were purified, and a pull-down assay was conducted to confirm the interaction between PuMYB21 and PuMYB54. The results showed that PuMYB21 interacted with PuMYB54 (Fig. 8c). Finally, a BiFC assay was conducted using tobacco leaves to verify the interaction between PuMYB21 and PuMYB54 in vivo (Fig. 8d). PuMYB54 was fused to the N-terminal fragment of yellow fluorescent protein (YFP) to form PuMYB54-YFP^N^, and PuMYB21 was fused to the C-terminal fragment of YFP as PuMYB21-YFP^C^. The results showed that a yellow fluorescence signal was observed in the onion cells coexpressing PuMYB54-YFP^N^ and PuMYB21-YFP^C^, suggesting that PuMYB54 physically interacted with PuMYB21 in the nucleus.

## Discussion

### The expression of PuPLDβ1 is related to peel browning during the refrigeration and subsequent shelf life periods

Cold storage is a valid method to extend the shelf life of postharvest fruits and vegetables, but it can cause some extent of CI^4^. Peel browning is a main CI manifestation in refrigerated “Nanguo” pears during its shelf life, affecting the quality and reducing the commercial value of the pears^4^. The biomembrane is not only a barrier that protects cells from injury but also a primary location for sensing cold signals^10^. An increasing number of studies have reported that one of the main causes of CI is changes in membrane lipid composition, structure, and metabolic processes, which ultimately result in the degradation of cell membrane lipids^10^. In bell pepper samples, the level of total membrane lipids declined notably because of CI^6^. The authors of this previous found that the percentage of PA was higher in the CI samples than in the fresh samples and that the content level and percentage of PC exhibited an extreme decreasing trend, whereas the content of other phospholipids, such as PE, PG, PS, and PI, decreased slightly. Here, we found that the content level of all the membrane phospholipids in “Nanguo” pears refrigerated for 120 d was significantly lower than that it was in the fruit stored for 60 d. Furthermore, the percentage of PA in the peel of the browning pears stored for 120 d was significantly higher than that in the short-term refrigerated pears. In addition, the content levels and percentages of PC and PE in the long-term-stored fruit were significantly lower than those in the short-term refrigerated samples. PLD can catalyze the hydrolysis of phosphodiester bonds and produce secondary messengers, such as inositol triphosphate, diester glycerol, acetylcholine, and PA, which can cause a series of secondary reactions by changing intracellular protein kinase K and Ca^2+^ levels, thus completing the cell response to cold signals^15^. Under cold stress, PLD activity increases significantly, which facilitates the process of membrane lipid metabolism. In the current study, the PLD activity level in the fruit refrigerated for 120 d increased significantly during the shelf life period compared with the pears stored for 60 d. These results suggested that low temperature stress stimulated an increase in PLD enzyme activity and increased the possibility of membrane lipid degradation, which might be one of the main reasons for the formation of peel browning. Plant PLD is composed of a family of heterogeneous enzymes with distinguishable catalytic and regulatory properties, endowing them with diverse physiological functions^34^. In cucumber, higher PLD activity at 2 °C was due to increased PLD mRNA levels^35^. The transcription level of *Arabidopsis* *PLDβ1* was significantly increased by bacterial and fungal pathogen infection^36^. A similar study was reported 10 years later, in which *PLDβ1* was found to be a negative regulator of salicylic acid-dependent resistance to bacterial Pst DC3000 but a positive regulator of the jasmonic acid-dependent pathway and resistance to the fungal pathogen *Botrytis cinerea*^37^. *PLDβ1* could also be induced by wounding stress^38^. The suppressed expression of rice *PLDβ1* inhibited the germination of rice seed^39^. In addition, the suppression of Os*PLDβ1* could also activate defense responses and increase disease resistance^40^. These results indicate that *PLDβ1* has a crucial role in plant growth, development, and the response to stress. In this study, the expression patterns of *PuPLDs* were investigated. The expression of *PuPLDα1* and *PuPLDα4* in the two groups of pears fluctuated during the shelf life period. The expression of *PuPLDδ* and *PuPLDζ2* in the long-term-refrigerated pears exhibited a downregulated trend after refrigeration. However, the expression of *PuPLDβ1* was significantly increased after transfer from long-term cold storage, and its transcription patterns were consistent with PLD activity. Thus, we speculate that *PuPLDβ1* has an important role in cold-induced membrane lipid metabolism and peel browning in “Nanguo” pears.

### PuMYB21 and PuMYB54 directly upregulate the expression of *PuPLDβ1*

Our research showed that low temperature can induce the expression of *PuPLDβ1* and promote the degradation of membrane lipids, thereby mediating the peel browning of cold-stored “Nanguo” pear. However, the mechanisms involved in how *PuPLDβ1* is regulated during cold stress remain to be studied. TFs are regulators that have important roles in many biological processes in plants by regulating spatiotemporal gene expression through recognizing specific DNA sequences in target gene promoters^41^. In this study, two candidate MYB TFs were identified that directly bind to the *PuPLDβ1* promoter and activate the expression of the *PuPLDβ1* gene. A comparison of the PuMYB21 and PuMYB54 amino acids with MYB proteins from other plant species revealed that PuMYB21 and PuMYB54 may be clustered into the typical R2R3-type MYB. Furthermore, phylogenetic analysis suggested that PuMYB21 and PuMYB54 shared a specific sequence similarity to PbrMYB21 of *Pyrus betulaefolia* and MdMYB54 of *Malus domestica*, respectively, indicating that PuMYB21 and PuMYB54 are putative MYB homologs of *Pyrus ussuriensis*. The overexpression of PbrMYB21 improved drought tolerance in *P. betulaefolia* by upregulating the expression of stress-related genes, including ADC, P5CS, and LEA5^42^. OsMYB30 is a cold-response R2R3-type MYB gene, and the overexpression of OsMYB30 increases cold sensitivity, whereas the osmyb30-knockout mutant showed increased cold tolerance. Moreover, OsMYB30 interacted with OsJAZ9, which had a significant role in suppressing the transcriptional activation of OsMYB30 and in repressing the BMY genes mediated by OsMYB30. This repression of BMY increased the content level of maltose, which might contribute to cold tolerance as a compatible solute^43^. In the current study, the expression patterns showed that PuMYB21 was significantly increased by long-term refrigeration and markedly boosted on the sixth day of shelf life after refrigeration. Similar to PuMYB21, the expression of PuMYB54 exhibited a continuous increase regardless of the previous length of refrigeration or subsequent shelf life. These results indicated that PuMYB21 and PuMYB54 were also induced by cold stress, and their transcriptional patterns were similar to those of *PuPLDβ1*. Considering these results, we speculated that PuMYB21 and PuMYB54 might bind to the *PuPLDβ1* promoter and regulate its expression, thereby mediating and participating in the peel browning in refrigerated “Nanguo” pears. The MYB *cis*-acting element contains a MYB recognition site with the TAACTG sequence, called MBS, in the target gene promoter^44^. Previous studies showed that many MYB TFs can recognize and bind to the TAACTG consensus sequence to modulate the transcription of their downstream target genes, such as *Glycine max* GaMYBJ1, *Oryza sativa* OsMYB2, *P. betulaefolia* PbrMYB21, and *Malus domestica* MdMYB1^45–47^. In the current study, sequence analysis showed that *PuPLDβ1* contains MBS in its promoter region. Furthermore, Y1H and EMSA assays demonstrated that the PuMYB21 and PuMYB54 proteins can specifically bind to the *PuPLDβ1* promoter by recognizing the MBS *cis*-element with the conserved TAACTG sequence; this result is consistent with that of previous studies. In addition, because the expression levels of PuMYB21, PuMYB54, and *PuPLDβ1* were upregulated during the process of cold-induced peel browning, we speculated that the occurrence of peel browning in refrigerated “Nanguo” pears was induced by the positive regulation of PuMYB21 and PuMYB54 on *PuPLDβ1*. To verify our speculation, GUS and dual-luciferase reporter assays were conducted. The results indicated that PuMYB21 and PuMYB54 can indeed activate the transcription of a reporter gene downstream of the *PuPLDβ1* promoter. These results were also verified by analyzing the expression of *PuPLDβ1* in overexpressed and silenced “Nanguo” pears and calli that have transiently expressed the *PuMYB21* and *PuMYB54* genes. These findings are the first to demonstrate that PuMYB21 and PuMYB54 can activate *PuPLDβ1* expression to mediate membrane lipid metabolism.

### The interaction of PuMYB21 and PuMYB54 promotes the upregulation of PuPLDβ1 expression

The regulation of gene expression by TFs is a complex process in which one or more TFs or other proteins may participate in coordinated expression. In banana, the expression of MaMYB4 alone significantly repressed the transcription of *MaFAD3-1*, *MaFAD3-3*, *MaFAD3-4*, and *MaFAD3-7*; however, this repression was further enhanced when MaMYB4 was coexpressed with MaHDA2, which interacts with MaMYB4^48^. Two MYB TFs, GmMYB76 and GmMYB177, were shown to enhance the freezing tolerance of *A. thaliana* by activating the expression of freeze-tolerance-responsive genes^49^. In this study, we found that the transcription level of reporter genes downstream of the *PuPLDβ1* promoter was enhanced by the simultaneous existence of PuMYB21 and PuMYB54. Therefore, we suspect that this phenomenon may be induced by the interaction between PuMYB21 and PuMYB54 or the overlapping individual effects of PuMYB21 and PuMYB54. To verify whether there is an interaction between PuMYB21 and PuMYB54, Y2H, BIFC, and pull-down assays were performed. These assays proved that there was indeed an interaction between PuMYB21 and PuMYB54. These results help us further understand the regulation of membrane lipid metabolism in refrigerated “Nanguo” pears. Furthermore, this is the first study to research the interaction between two MYBs during cold storage in fruit. However, the specific synergistic model of PuMYB21 and PuMYB54 needs further study.

In this study, we identified two MYB TFs, PuMYB21 and PuMYB54, expressed in the peel of browning “Nanguo” pears after refrigeration. The two MYB TFs belong to the R2R3-MYB family and are closely related to PbrMYB21 (XM_009359681.2) from *Pyrus* × *bretschneideri* and MdMYB54 (XM_017337313.2) from *Malus domestica*. They bind to the promoter regions of *PuPLDβ1* and coregulate its expression. Correlating with the increased expression of PuMYB21 and PuMYB54, the expression level of *PuPLDβ1* was elevated during the pear refrigeration and subsequent shelf life periods. Our discovery led to new regulatory targets and metabolic pathways of PuMYB21 and PuMYB54 proteins and complemented the current understanding of the coregulatory network of the membrane lipid degradation mechanism, providing a new perspective for revealing the molecular mechanism of membrane lipid degradation-induced peel browning in cold-stored “Nanguo” pears. It remains to be investigated whether other TFs bind to the *PuPLDβ1* promoter and regulate membrane lipid degradation in plants. The members of these TF families can participate in more metabolic pathways, and these results suggest that different TF families may be recruited in the same way to commonly regulate peel browning of “Nanguo” pears through different metabolic pathways.

## Materials and methods

### Plant material and treatment

“Nanguo” pears were harvested on September 11, 2018, at an orchard located in Anshan, Liaoning Province, China. The picked fruits were transported in <3 h to the laboratory and then selected based on uniform color, size, and absence of pests and damage induced mechanically or by disease. The fruits were randomly allocated into two triplicate groups, each with 300 fruits, which were placed at room temperature (20 ± 1 °C) for a 4-d pre-ripening period and covered with newspaper to prevent water loss^50,51^. After pre-ripening, all of the fruits were precooled at 0 ± 0.5 °C for 24 h and then placed in reusable plastic boxes lined with 0.04-mm-thick polyethylene bags. There were 50 pears in each plastic bag, and each bag was tied and stored at 0 ± 0.5 °C with 80–85% relative humidity (RH). After storing for 60 and 120 d, the fruits were moved to room temperature for the shelf life study (20 ± 1 °C, 80–85% RH, natural light). Fifty fruits were used to evaluate peel-browning indexes and browning incidence. The peel tissues of the remaining fruits were frozen in liquid nitrogen every 3 d and stored at −80 °C until the biochemical analysis was performed.

The calli of “Nanguo” pears were used for *A. tumefaciens* infiltration. The calli were cultured on solid Murashige and Skoog (MS) medium containing 2.0 mg of 2,4-dichlorophenoxyacetic acid and 1.5 mg of 6-benzylaminopurine at 28 °C in the dark (Supplementary Fig. S2).

### Incidence and index of peel browning

The incidence of peel browning was characterized as the proportion of browning fruit to total fruit. The value is expressed as a percentage.

The browning index (BI) was calculated as previously described^5^. The severity of browning was assessment based on the following four-grade scale: 0 = no peel browning, 1 = slight (browning area < 1/3 of the total area), 2 = moderate (1/3 < browning area < 2/3 of the total area), and 3 = severe (browning area > 2/3 of the total area). The result was calculated using the following formula: BI (%) = Σ [(browning scale) × (number of fruit at that scale)]/(3×total number of fruit) × 100.

### Membrane phospholipid content

The extraction of membrane phospholipids was performed as previously described^16^. The solution containing membrane phospholipids was evaporated under nitrogen. Membrane lipid extracts were dissolved in 1 mL of chromatographic grade methanol, and then detection was conducted using automated electrospray ionization-tandem mass spectrometry as previously described^52^.

### Determination of phospholipase D activity

The activity of PLD in the peel tissue was determined as previously described^53^. The pellet containing PLD was dissolved in 1 mL of acetone, and the activity of PLD was determined using a spectrophotometer (TU-1810 DSPC, Beijing Puxi Instrument Co., Beijing, China) at 520 nm. One unit of PLD activity was defined as a change in absorbance of 0.01 per hour at 520 nm. The specific PLD activity was based on the protein mass; it is expressed as U mg^−1^ fresh weight.

### RNA extraction and RT-qPCR analysis

Total RNA was extracted from the “Nanguo” pear peel using the RNApure plant kit (DNase I) (CW0559S, CWBIO, China) following the manufacturer’s instructions. Total RNA concentration and purity were quantified using a Microplate reader (Eon; BioTek, USA) at OD_260_/OD_280_ and OD_260_/OD_230_, and the integrity of the total RNA was detected using 1.0% agarose gel electrophoresis. The RNA obtained was used as the template to synthesize cDNA using a HiFiScript cDNA synthesis kit (CW2569, CWBIO, China) according to the kit instructions. RT-qPCR was performed using the UltraSYBR mixture kit (CW0957M, CWBIO, China) in 20 μL of volume, according to the kit instructions, on a QuantStudio™ 5 Real-Time PCR system (ThermoFisher, USA) with the following protocol: denaturation step of 94 °C for 5 min, followed by 40 cycles of 95 °C for 10 s and 60 °C for 1 min. Dissociation curves were generated for gene primers to detect the presence of nonspecific amplification. The expression of the target gene was normalized to that of the internal reference gene (*PuActin*) using the 2^−ΔΔCt^ method (the comparative Ct method). The specific primer sequences that were designed by using Primer Premier 6.0 software and synthesized by GENEWIZ Biotechnology Synthesis Lab (Jiangsu, China). The primers used are listed in Supplementary Table S2. A total of three biological replicates were included.

### Promoter isolation and analysis

A NuClean Plant Genomic DNA kit (CW0531S, CWBIO, China) was used to extract genomic DNA from “Nanguo” pear peel tissue following the manufacturer’s instructions. The *PuPLDβ1* promoter fragment was isolated according to the sequence (ID:103966461) in Chinese white pear (primers are listed in Supplementary Table S3, and the promoter sequences are listed in Supplementary Table S4). The conserved *cis*-element motifs of the *PuPLDβ1* promoter sequence were predicted using PlantCARE online tools (http://bioinformatics.psb.ugent.be/webtools/plantcare/html/)^54^.

### Yeast one-hybrid (Y1H) assays

To construct the prey and bait vectors, the full-length CDSs of the TFs were cloned (the primers are listed in Supplementary Table S3, and the sequences are listed in Supplementary Table S4) and inserted into the pGADT7 vector, and the promoter of *PuPLDβ1* was inserted into a pAbAi vector. The Y1H assay was conducted using a Matchmaker Gold yeast one-hybrid system (Clontech) as previously described^55^.

### Electrophoretic mobility shift assay (EMSA)

The full-length CDSs of PuMYB21 and PuMYB54 were inserted into a pET-SUMO vector to construct expression vectors, which were introduced into *Escherichia coli* strain BL21 (DE3) cells. To purify the proteins, the cells were incubated in 0.5 mM isopropyl-β-d-thiogalactopyranoside (IPTG) for 3 h at 30 °C.

The EMSA assay was performed using a LightShift chemiluminescent EMSA kit (20148, ThermoScientific, Illinois, United States) as previously described^53,56^. A small part of the *PuPLDβ1* promoter fragments with MYB-binding sites was synthesized and labeled with biotin probes (Gene Create). The EMSA assay was performed as previously described^55^.

### β-Glucuronidase (GUS) analysis

The *PuPLDβ1* promoter was inserted into the pBI101 vector to form the reporter construct. The CDSs of PuMYB21 and PuMYB54 were constructed and inserted into a pRI101 vector to form effector constructs. Both the reporter and effecter constructs were transferred into *A. tumefaciens* strain GV3101 and used for the coinfiltration of tobacco leaves. After 3 d, the infiltrated leaves were used to measure the activity of GUS as previously described^57^.

### Dual-luciferase reporter assays

The promoter of *PuPLDβ1* was constructed into the double-reporter vector pGreen II 0800-LUC to form the reporter construct. The CDSs of PuMYB21 and PuMYB54 were constructed in pGreen II 62-SK vectors to form effector constructs. The reporter and effector constructs were transferred into *A. tumefaciens* strain GV3101 and used for the coinfiltration of the tobacco leaves. After 3 d, the activity levels of the LUC and REN luciferases were detected using a Dual-Luciferase^®^ reporter assay system (E1910, Promega, Madison, WI, USA) and analyzed with a Luminoskan Ascent microplate luminometer (ThermoScientific, USA) following the manufacturer’s instructions. The LUC activity level was normalized based on the REN activity level. The binding strength of PuMYB21 and PuMYB54 to the promoter of *PuPLDβ1* was calculated based on the LUC-to-REN ratio^58^. At least six biological replicates were included for each combination.

### Yeast two-hybrid (Y2H) assays

The Y2H assay was conducted using the Matchmaker Gold yeast two-hybrid system (Clontech, USA)^55^. To identify whether PuMYB54 interacts with PuMYB21, the CDS of PuMYB54 was inserted into a pGBKT7 vector as bait. pGADT7-PuMYB21 was the prey protein. The bait was transformed into Y2H yeast cells and grown on synthetic medium without tryptophan (Trp) and containing 200 μM aureobasidin A and 20 mg mL^−1^ chromogenic substrate X-α-gal, which turns blue upon transactivation.

A toxicity test indicated that the full-length PuMYB54 protein does not cause toxic effects in the yeast host cell (data not shown). The Y2H yeast strains were cotransformed with PuMYB54 + PuMYB21, PuMYB54N + PuMYB21 or PuMYB54C + PuMYB21. PuMYB54 + pGADT7 or pGBKT7-53 + pGADT7-T was used as a negative or positive control.

### Bimolecular fluorescence complementation assays (BiFC)

BiFC assays were conducted in onion tissue to confirm the interaction between PuMYB54 and PuMYB21^32^. The CDS of PuMYB54 was fused to the N-terminal fragment of yellow fluorescent protein (YFP) in a 35S-pSPYNE-YFP^N^ vector to generate PuMYB54-YFP^N^, and the CDS of PuMYB21 was fused to the C-terminal fragment of YFP in a 35S-pSPYNE-YFP^C^ to generate vector PuMYB21-YFP^C^. The transformed *Agrobacterium* was mixed (1:1, v/v) and used to infect onion epidermal cells. After 3 d, the YFP fluorescent signal was detected with a laser scanning confocal microscope (TCS SP8-SE; Leica, Wetzlar, Germany).

### Pull-down assays

The CDS of PuMYB54 was recombined into a GST-tagged PGEX-6P-1 vector, and the CDS of PuMYB21 was inserted into the His-tagged pET-SUMO vector^55^. The reconstruction vectors of PuMYB54-GST and PuMYB21-His were transformed into BL21 to induce proteins. The pull-down assays were performed using a His-tagged protein purification kit (Clontech, Palo Alto, CA, USA) according to the manufacturer’s instructions. The eluted samples were detected by western blotting using HIS and GST antibodies (Abcam, Cambridge, UK).

### Vector construction and the generation of transgenic “Nanguo” pears and calli

The CDSs of PuMYB21 and PuMYB54 were constructed into the pRI101 vector to form PuMYB21-OE and PuMYB54-OE vectors. For RNAi constructs, the interference fragments of PuMYB21 (386 bp) and PuMYB54 (387 bp) were obtained using the primers (Supplementary Table S5) and inserted into a pCAMBIA2301-ky vector to form PuMYB21-Anti and PuMYB54-Anti vectors (interference fragments are listed in Supplementary Table S6). The vectors were transformed into *A. tumefaciens* strain GV3101. The preparation of the infection suspension was performed as previously described^55^.

To overexpress or silence PuMYB21 or PuMYB54 in “Nanguo” pears, 100 μL of infection suspension was injected into pears at a depth of 0.5 cm. Ten fruits were infiltrated with each construct. Three fruits were used as biological replicates. To overexpress or silence PuMYB21 or PuMYB54 in “Nanguo” pear calli, the “Nanguo” pear calli were dipped into the infection suspension for 25 min at room temperature for transfection. The infected calli were then grown in the dark at 28 °C on solid MS medium containing 2,4-D and 6-BA for 8 d and subsequently used for RNA extraction, as described above. Each transfection was repeated three times for three biological replicates.

### Bioinformatics analysis of PuMYB21 and PuMYB54

The full CDS sequences of PuMYB21 and PuMYB54 were amplified according to the accession numbers XM_009359681.2 and XM_017337313.2, respectively. The comparisons of the amino acid sequences for homologous *PuMYB21* and *PuMYB54* were performed using ClustalX 1.83 (http://align.genome.jp/). A phylogenetic tree was constructed using the neighbor-joining method in MEGA5.0.

### Subcellular localization of PuMYB21 and PuMYB54

To investigate the subcellular localization of *PuMYB21* and *PuMYB54*, the full-length coding sequences (CDSs) of *PuMYB21* and *PuMYB54* without terminate codons were inserted into the pRI101-eGFP vector using *Nde*I and *Kpn*I restriction enzyme sites to form the fusion expression vectors PuMYB21-eGFP and PuMYB54-eGFP under the regulation of the cauliflower mosaic virus (CaMV) 35S promoter. The fusion expression vectors were transferred into *A. tumefaciens* strain GV3101 and used to transform onion epidermal cells as previously described^59^. Laser scanning confocal microscopy (TCS SP8-SE; Leica, Wetzlar, Germany) was used to detect fluorescence signals.

### Statistical analyses

All experiments were repeated at least three times. Statistical analyses were performed using SPSS 19.0 software (IBM Inc., Chicago, IL, USA). Two groups of data were compared using Student’s *t*-test, and a difference was considered to be significant when *P* < 0.05.

## Supplementary information

Supplementary Information
