# Supplementary material for: PuMYB21/PuMYB54 coordinate to activate PuPLDβ1 transcription during peel browning of cold-stored “Nanguo” pears
Source: Hortic Res. 2020 Sep 1;7:136. doi: 10.1038/s41438-020-00356-3 (PMC7459126; doi:10.1038/s41438-020-00356-3)
Supplement: Supplementary file 1 — Supplementary Information [file 41438_2020_356_MOESM1_ESM.docx]

**Table S1** **transcription factor expression in response to long-term refrigeration.**

| Gene names | Log2 (fold change) |
| --- | --- |
| PuMYB4 | -4.2 |
| PuMYB6 | -2.3 |
| PuMYB21 | 1.76 |
| PuMYB24 | 2.1 |
| PuMYB44 | -1.2 |
| PuMYB54 | 1.75 |
| PuMYB66 | 5.3 |
| PuMYB84 | 3.6 |
| PuMYB86 | 4.7 |
| PuMYB91 | -1.21 |
| PuMYB306 | -3.22 |
| PuMYB1R1 | -2.43 |
| PubHLH13 | 1.45 |
| PubHLH15 | -2.12 |
| PubHLH21 | 2.44 |
| PubHLH66 | 1.43 |
| PuWRKY20 | 2.65 |

**Table S2 RT-qPCR primer list**

| Gene | Forward primer (5′-3′) | Reverse primer (5′-3′) | Product size (bp) |
| --- | --- | --- | --- |
| *PuPLDβ1* | GCGGAAGTTCCTTGCCTGAGTG | GAGCCTCACGGATTGCCTTGAC | 196 |
| *PuPLDα1* | AGAGTCGGCATCTGTTCAGGCA | CCTCCCGATTCCCAAGGCAGAA | 149 |
| *PuPLDα4* | AGGCGGATGAGCACAGAGGATT | GCTACCATCTTCAATGCCACACC | 169 |
| *PuPLDδ* | AGATCATCCGCTCCTCCGACTC | TGCCTACAACCAACGCCAACG | 149 |
| *PuPLDζ1* | GCCAACTTGTCCCGATTCATCTGT | ACCAGTAGGTCCTGATACGCCA | 145 |
| *PuMYB21* | GGACTGCTGAGGAAGATGGATTG | GCAAGCCTAGCCACAGAGTTCC | 167 |
| *PuMYB54* | TGGACCTCATAACTGGAACGCAA | GACGCTCTTCTTCCTCTTCTGTG | 156 |
| *PuActin* | TTGGGATGGGTCAGAAGG | CTGTGAGCAGAACTGGGTG | 186 |

**Table S3 Primers used for the cloning of *PuPLDβ1* promoter and TFs CDSs**

| Gene | Forward primer (5′-3′) | Reverse primer (5′-3′) | Product size (bp) |
| --- | --- | --- | --- |
| *PuPLDβ1p*-1 | AAGCTTTCGAGTGTCGACAG | ACGCAATTTGTGTACATGTCC | 700 |
| *PuPLDβ1p*-2 | TATTGGGGTTGGGGCGTGTC | CTAGTGAGCACTAGGACTCC | 894 |
| *PuPLDβ1p*-3 | CTGTTGGTCTTAACCAACC | CATGGTGGGTGGGTTTGACT | 1146 |
| *PuPLDβ1p*-4 | GGGTAAAACTTGCCTTAATAG | TATCTAGTGATTGCTATGTG | 736 |
| *PuMYB4* | ATGGTTAGAGCTCCTTGCTG | TCAAATTCCTAACAATTCTGG | 804 |
| *PuMYB6* | ATGGGGAGGAGTCCATGTTG | TTAGACAATAACATTTTCTTG | 885 |
| *PuMYB21* | ATGTATTTGGGGATGATGG | CTAGCTTTGCCTGGTGGTGT | 720 |
| *PuMYB24* | ATGCCAGCGACGGAATACTCG | TCAATTGGCAATTGATTTCGATG | 1218 |
| *PuMYB44* | ATGGAAGCATTGAATATTTGC | TCAAAGAAGCCCCGAAGGCTC | 717 |
| *PuMYB54* | ATGTGCACTAGAGGCCATT | TTATGAAGAAATCCCATTATT | 813 |
| *PuMYB66* | ATGGACGGTGGGAATGATT | TCATCCTGAGTAGGGTCCGAT | 678 |
| *PuMYB84* | ATGGGGAGAGCTCCTTGCTG | TCAGTAGTACATCATGACC | 960 |
| *PuMYB86* | ATGACGGCCCCAAACGACG | CTAGGTAGTGGCAGCTGCTTG | 1086 |
| *PuMYB91* | ATGAAGGAGAGACAGCGTTG | TCACCGGCCATTAGGCTCGAC | 1062 |
| *PuMYB306* | ATGGGAAGGCCACCTTGCTG | TCAAAACAAGCCTGCACCA | 1143 |
| *PuMYB1R1* | ATGTCGTCCGGCACGTGC | TTAAGCGACGCTGATCACGCC | 903 |
| *PubHLH13* | ATGAAGATTGAGATGGGTTT | CTACCCGACTGATGACAACG | 1842 |
| *PubHLH15* | ATGGACTACGACTACTTACT | TTAACCTGATTGATGGTTTTCA | 1566 |
| *PubHLH21* | ATGAGAACCACCGCAAAAGG | CTATGTCTTGAGGCGCTTATG | 1005 |
| *PubHLH66* | ATGCAGCCTTGTAGTAGAGA | TCAGGGCTTGGAGACGGAGG | 1407 |
| *PuWRKY20* | ATGGACTCGATCGCACTCGAC | TCACGGACCTGTTAGTACTCTTC | 1761 |

**Table S4 Sequence of *PuPLDβ1* promoter and TFs CDs sequences used in this study**

| gene name | sequence |
| --- | --- |
| *PuPLDβ1_pro_* | TCGAGTGTCGACAGCACGTGCCTACCCTGATATTTGGGGATATTGGGGTTGGGGCGTGTCAAAATTTCTTTCGCAAACACTTTCTACATGTACTAGGATCTGCCCGATCGGTGGATAACTATGCCTAACTGTGGGTAAAACTTGCCTTAATAGGTTTAATCTTTGCCCGATCACTTGTATTATTTGTTTATTTTCAATTTATTATATTTTCATGCAATTTAGTATGGTTTAATTAATATTTTGCAAGTCTTGACAGCTTCTTACTTTCACCTTTTTATTGTACTTTGTTTCGTTTTACTTACTGTTTTTACATTTATAACAAAGATTGCAAACTGACTTAGATCACTTTTGATCAGTTTGTGGTTCTCTTCTTAGCATCAATATAAAAGAAAATAAGAGTCATTGTCACACCTTGTCACCAAGGCATAAAAAGAACTTAACATGGATTTAATCCTTCCATGGAACAATTTTTGATAAGAAGTTCAGTTTACTTGGGGCGCAGGCAGACAAACTCAACAAATACTTAATAAACAATCAATCAGTTGGTCTTAACCAACCATGGCACACCTGAAAGCAATAAAATTGTTTATAAGAGCCTAAAGACCTACTCAAGGCTTTGACAAGGCACCTTAAACTAACGCAAAGTTCTACACTCATGTAAAAAACGTGTGGGGATTGACCATCCAACACTAGTAGTGTT |
| *PuMYB4* | ATGGTTAGAGCTCCTTGCTGCCAGAAGATGGGTTTGAAGAAAGGGCCTTGGACTGCAGAAGAAGATCACATACTCGTCACTTACATTCAGCAGCATGGCCATGGAAACTGGAGAGCTCTCCCAAAACTAGCTGGTTTGTTACGGTGTGGAAAGAGTTGTAGACTCAGATGGACAAACTACTTGAGGCCAGATATCAAAAGAGGGAATTTTAGCAGGGAAGAAGAAGACGCCATCCTCAACTTACATCAAATGTTAGGAAATAGATGGTCACAAATCGCAGCGAGATTGCCTGGTCGCACAGACAATGAAATAAAAAATGTATGGCACACCCATTTAAAAAAAAGGCTGAAATCGAACCAAACCGAATCAGATTCTAGGGCACATTTCTACTCTAGAAAAACGGAGCAAGAGCTAGAGCCCATTGATGATTCTTCACATTGTGCAAAATCTGATAGCTTGGATTGTGTGCCTGTTTCGCCGGCTCGATGTACTTCAAGTAACACGTCATTTGTCACGACCAACGAGGACAACAACAACAACATTAGTAACAATGTATGCGTGACATTTGATGATTACCAAGCAAATTTACCAGAACCGGATGATAGTTTTTGGTCGGAAGTTTTGTCAACTGAGTATTATGAAGAGACGGTGAATGAGTTTCAGGCATTTGGTGGTGACCCACAAGTGAATATGGGGCCTACTGATGGTGGTTTTAGTTTAGACATGTACAATGACAACAACATGGACTTTTGGTTTAATATATTCTCAGGAGCTGAGGAAATTCCAGAATTGTTAGGAATTTGA |
| *PuMYB6* | ATGGGGAGGAGTCCATGTTGCTCCAAGGAAGGACTCAACAGAGGAGCCTGGACTGCCTTGGAAGATAAAATTCTAACAGCTTACATTAAAGCCCATGGAGAAGGCAAATGGAGAAGCCTTCCCAAAAGAGCCGGTCTAAAGAGATGTGGTAAAAGTTGCAGATTGAGATGGTTAAACTATCTGAGACCAGACATAAAGAGAGGCAACATTTCAGGTGATGAAGAGGAACTCATTGTTAGACTCCATAACCTTCTTGGTAACAGATGGTCGTTGATAGCCGGAAGGCTACCGGGGCGAACAGACAATGAAATCAAGAATTACTGGAACACAACTTTGTGGAAGAAATCGAAAGCCGATTCTCCTTCTGGATGCTCGAAAGAAACTTCTCAACATCCAAGCAAATCCGTAGTGAAAAAGAAAGATGTCGAGTCCAAAACAACATCAACTGCAGCTGCTAAACCTCTAGTAATAAGAACCAAGGCCACTAGGTTGTCCAAAATTTTAGTCCCACAAAAGATTCCTAGCGATGAAAATTATACAGCAGCTGCCGCAAACCCATTAGAGCTGCAATCTCATCAAACCCAATTGGCGGAACAAGGCGGAAGCACCGAAGAGTTTCCGAGGACTAATGCAGACGACCGCAGCAACATCTTGAAGAACTTTGGCTGCGATGAGGACGACATTGATGTGAAGGGAGATCAATACTGCAATGAATTTCAGTCGCTCGACTCTATACCGTTGGATGAGGCAATGATCAACGACGGCTGCTGGACGGTTGGAAACGGTTGCGAGCTGGAGGACTACGGTGCCTCATTGGATTTAGATTCTTTGGCATTTTTGCTTGACTCTGAGGATTGGCCATCCCAAGAAAATGTTATTGTCTAA |
| *PuMYB21* | ATGTATTTGGGGATGATGGCAGGAGGACATCAGAGGGGTTGGGGGATGATAGAAGATGAGGCTTGGAGAAAGGGACCTTGGACTGCTGAGGAAGATGGATTGCTCATTGAGCATGTGAGGCTTCATGGTGAAGGCAGATGGAACTCTGTGGCTAGGCTTGCAGGGCTGAAAAGGAATGGAAAGAGCTGCAGATTAAGGTGGCTGAACTATTTGAGGCCAGAACTTAAAAGGGGGCACATAACTCCTCATGAAGAGAGCATCATTCTAGACCTACATGCTAGATGGGGAAACAGGTGGTCGACAATTGCACGAAGTTTGCCTGGAAGGACTGACAATGAGATCAAGAACTACTGGAGGACTCATTTCAAGAAAAAGGCGAAAGCACCTTCTGATGCCTCCGAAAGGGCCAAAACCCGCCTCTTAAGGAGACATAAACCCCATAATCAGCAGCAACAACAACAAAATTTGCAAGTGGATCAAGAAGTGAAAAGAATCATGTCCTTATTGGACGAAAATGAGACTAAACTGCCGTTATATTGGCCTCACGCAGCTGATCATCATCAGGAGCAAGGCTTCTTCTATTCCATGCTCGATGGCAATATTGTCAATGTGTCTATGCCTGAAGCTGCCTCCAGCAATGAGGTCAATTTTCTGTGGGATGGTTTGTGGAACTTAAATGATGTCCATGCAAACTTTATTAACACCACCAGGCAAAGCTAG |
| *PuMYB24* | ATGCCAGCGACGGAATACTCGTCAGCTGGTTCCAATCAAGTCGGAAGGTCAATCTTCAGTCTCAGACGCGATCAGTTGAATTCAATGGAAAGTCACGGCCTTTCAGGTGGCAGCGGTAGTGGCGGATTGGACCTCGATTCCTTCCAAACCCACGTCGCCAATTGCTTCCACAACCTCTCCTCCTCCGCCCACGACCTCCTCTCCCTCCCATGGCTCCGAGACCTCCTCGAATCCTTCATCCGCATACACCAGGAGTTCAAAACCCTCCTCATCCTCTCCTCCACCAAAACCCACATCTCCAAACCCACCGTCGACCGCTACATCTCCGACTATTTCGAGCGCCACGTCAAGGCCCTCGACGTCTGCAACGCCATCCGCGACGGAATGGAGCAGATGCGCCAGTGGCTCAAGCTGCTCGACATCGTCCTCGTCGCGCTAGACCGGGACAGGACGCTCGGCGAGGGCCAATTTCGCCGGGCGAAGAAGGCCCTCGTCGACCTAGCCATCGCAATGCTCGACGACAAGGACTCCTCCAACACCACCATAGCCCACCGGAACCGCTCCTTCGGCCGAAACAACAACACAAAGGATCATCAGCACCAGCAGCACCGGTCGCTCGGCCACTTCCGATCTCTGTCCTGGAGCGTCTCACGGTCGTGGTCGGCCGCCAGGCAGCTCCAAGCAATCGGGAACAATCTATACGCCCCGAGGCCCAATGAAAATTTGGCCTCCAGTGGGCTGGCCCCGGCGGTTTTCACAATCAATTCGGTTTTGCTGTTCGTAATGTGGGCACTGGTGGCGGCGATACCCTGCCAGGACAGGGGATTGCAGGTGCATTTCAACGTGCCGAGGAGCTTCGCGTGGGCGGGGTCAATGCTGTCTCTGCACGAGCGGATTATGGACGAGTCGAAGCGGCGGGACAGGAGGAATGCCTGCGCATTGTTGAAGGAGATTCATCAGATCGAACGGTGCTCTCGGGTGATTGGCGATCTGGCTGACTCCGCGCACTTCCCGCTTGCGGAGGAGAAGGAGAGGGAGGTGAGGCAGAGAGTGCGGGAACTGTCGAGCCTCTGCCAGGGGATTAAGGAGGGGTTGGATCCTCTGGAGCGGCAGGTCAGGGAGGTGTTCCATGGAATTGTAAAAAGCCGAACGGAGGGGATGGACTCGTTCGGAAGAACGAATAACCCTGAATTATCATCGAAATCAATTGCCAATTGA |
| *PuMYB44* | ATGGAAGCATTGAATATTTGCTCGTCTTCTGCTTCGTCTTCCGACACGTCCTCGTCGGAGTCTTCTCTTCCGAGAAATCCCAATAAGCCCGAACGAATCAAGGGCCCGTGGAGCGCTGAGGAAGACCGGGTCTTGACCCGGCTCGTCGAGCGCTACGGGCCTAGAAACTGGTCATTGATAAGCCGGTATATAAAGGGACGGTCCGGGAAGTCGTGCAGGCTAAGGTGGTGCAACCAGCTGAGCCCGAGCGTCCAGCATCGATCCTTTTCGCAGGCCGAGGACGAGACCATCTTGGCCGCACAGGCCCGGTTCGGAAACAGATGGGCCACCATTGCGCGCCTGCTTCCGGGTCGGACCGACAACGCGGTGAAAAATCACTGGAACTCCACGTTGAAGCGGAGGGTCAGGGGAGATCAACTAACGGAGGGTGGCAGTTTTCTTGGCGCCGGCGGCAATGTGGGTAGCAATGAGGGGATGAGCACGAGTTCGGTTTCCGGATCTTTGGTTAATGGGTCGATGGAGTTTGACCCGTTGACGGAGTTGACTTTGGCACCGCCGGGGATTGGTAGCGGAAGCGGCCGGGCGATGGTGGCGGAGCAGCGGAGGAATAATGAGAGTGTGCCGGCGGGGTTTTGGGACGCGATGAGGGATGTGATTGCCAGGGAGGTTAGAGATTACGTGACGACAACATTTTCAGAGCCTTCGGGGCTTCTTTGA |
| *PuMYB54* | ATGTGCACTAGAGGCCATTGGAGGCCAGCTGAAGACGAGAAGCTTAGGGAGTTGGTGGAACGATATGGACCTCATAACTGGAACGCAATAGCAGAAAAGCTCCAAGGAAGATCAGGAAAAAGCTGTAGGTTGAGATGGTTCAATCAGTTGGACCCAAGAATCAATAGGAACCCCTTCACAGAAGAGGAAGAAGAGCGTCTCTTAGTTTCTCACCGCATTCATGGAAATAGATGGGCTGTTATTGCAAGGCTTTTCCCTGGACGTACCGATAACGCAGTCAAGAACCATTGGCATGTCATCATGGCACGCAGATGCAGAGAAAGATCTAGGCTTAATCACTCCAAATTAATAAGGAATACTTCTACTGCTCAAACCTCGTTCATGAGTATCATTACTAACAATAATGAACCAAAATCCTCTTCAAAACAAGACCATCCCCTGCAGATCAATTGCGATATCGGCACATGGAACCTGGATTCATATAATTATATTGGGAAATACTACTCATGCAGCGACAGAAATTATCATCAATATCGGCCAGCTTTCACTCATAACCCTCCTCCTCCATTTCCAAAAGAGTTCTATCCCAATACCTGCAGTGCCACAATGCACCAAGATCATAATAAAGATCAGCCAATTGAGTTTTACGATTTTCTCCAAGTAAATACTGAGTCTAATGGAAGTGAAGTGATAGATAATGCAAGAAAGGAGGAGGAGGAGGTGGATCAGGAAGGTACGGGACAACAGAAAAGCAACGTGGCCGGTCTTCCTTTTATCGATTTCTTAGCTGTTAATAATGGGATTTCTTCATAA |
| *PuMYB66* | ATGGACGGTGGGAATGATTATAAGAAAGGTCTTTGGACAGTGGAGGAGGACAGAATTCTGACGGATTACATCGGGGTGCATGGCAAAGGGAAGTGGAATCGTGTCAATAAAGTCACAGGATTGAAGAGGAGCGGCAAAAGTTGCAGATTAAGGTGGATGAATTATCTAAGTCCCAGCGTGAAGAGAAGTGACTTCTCAGAGGAAGAAGATGATCTCATCATTCGACTCCATAACCTCGTTGGCAACAGGTGGTCTTTGATTGCTGGCCGTGTTCCTGGGAGAACTGACAATCAAGTGAAGAACCATTGGAATAGTCATTTGAGCAAGAAGCTCGGTGTCAATTCAAAAAGAGGAAAGACAAAAGCCAAGACTTATTCCGACTCAAAACGAGCAAAAAATAATTTCTGCACACCCTCATCAGAGTCAAACTCCGAGTTGCAATCACTTCCCAATTCCGATTCCAATTCCAATATTTCTGGTGATCATGAAGCTGCAGCTGCCATTGATGGATTTGATGAGGAGATGATGAACATCCAGGACCACATTGGCTCTAAAAGTGAGGTTGGTTTTAGTGGCATGTCGGAGGCGACGATGATGAGTAATGACTACTCATTTTGGTTTCACAATGATGACCTTAATCCATATGATCCCTTATTTATCGGACCCTACTCAGGATGA |
| *PuMYB84* | ATGGGGAGAGCTCCTTGCTGTGACAAGGCAAATGTGAAGAAAGGACCGTGGTCACCTGAAGAAGATGCAAAGCTGAAAGAGTACATAGAAAGATATGGAACTGGAGGGAATTGGATTTCTCTTCCACAGAAAGCTGGTCTTAAGAGATGTGGGAAGAGTTGCAGATTAAGATGGCTAAACTATCTCAGGCCCAACATTAAACATGGTGAATTTTCTGATGAAGAAGATAACATAATCTGCAACCTCTTTACTAACATTGGAAGCAGGTGGTCAATTATAGCAGCTCAGTTGCCAGGCCGGACTGACAATGACATAAAAAACTACTGGAACACTAAGCTAAAAAAGAAGCTTATGGGCATAAGCATTCTCCCATCCCAGCGGCTAAAATCTCACTCGTCATCATACCAAGGGAGCAACACCAGCACTAGCTATTACACACAAACCAGGTCTTTCACCAACACTTTGGATCCCATTTCTTTTTCACAAAGTCTCATGAGCAGCACTTCTACTAATGCTGCTGCTGCTTCTGCACTCCAAGTCCCCCAAGAGAGCTTTGTGGGGAGACACATGCATCAGCAGCATTACCAAGTCAAAGATAACATCTTGATGTTTGGAGGTGAAGCAAGTTGCTGCTCTTCTGATGGGAGTTACAGCAATCATCAGATCAGAGAAAGAGAGTATGAATATGGTGGTGCTTATGGCGGCGGTGGCGGCGGTTCAACTAATGGGGAGGTGAAGTTACACATGGGTTTAAAAAATTATAGTTGTTTTACTGGGTTTGGAGGAGAGCAGAAAATTATGGAGAATAATTCCAGTGGAAATAGTGGGATGTGGGAAGATCAAGCACGATTAGACTATGGACTTGAAGAGATTAAGCAGTTAATTAGCAGTACAGGTAGTTGCAACAACTTTTTGTTTGATGAAAACAAGACACAGGAAAAGGTCATGATGTACTACTGA |
| *PuMYB86* | ATGACGGCCCCAAACGACGCCGTCCCCAAAGAAGCCGACGACCGCCCCAGCACCGAGGCCGAGTTGAACGAGGGCGCAGTGCCCGGCGGGAAAGTGAGGGGACCGTGGTCGCCCGAGGAGGACGCGGTGCTGAGCCGGCTCGTGGGCAACTTCGGGGCGAGGAATTGGAGCCTGATCGCCCGAGGAATTCCCGGACGGTCTGGGAAGTCGTGCCGGCTGAGGTGGTGTAATCAGCTTGACCCCTGCGTCAAGCGTAAACCCTTTTCTGAGGAAGAAGACCGTATTATAGTTTCAGCACATGCTATCCATGGGAACAAATGGGCAGTAATTGCAAAGCTTCTTCCAGGTAGAACAGATAACGGAATCAAGAACCATTGGAATTCTACACTAAGGCGCAAGTGCTTTGATAAAGGAAGGTTTAATACTGGACATGGGGAAATGATGGAAGATGACACCTTTGACAGAAAAAATGCATCCTCAGAAGAAACCCTGTCAGTTGGGAATATCAGTTCATTCAAGACTCATGAAGGAAGAGAGGTCTTGATGGAAAATAGACCAAGCCAGTTCGACGTAAGAAGTCATGCAAAGGAGGGTTCTGGCGCTGCCGAATCAAAGCACAATTCTACTCTTATTGCCGAGCCAAGTGACCATCCAACTCTCCAGTCCACCATTTGTCGTCCAGTGGCACGTGTTAGTGCTTTTAGTGTTTATAACCGTCCAAGTGGTCCAGCAAATGCTTCATCGTTTTCAAGGACAGTCCCAAGTCATAGCCCTTTGGTCCCAATAACTAAACTAGATTTCGGCTTTGACAACTTCCTTGAAGGTGCATGCAATGAGCCTATGGTTCCTCAACGCTGTGGCCACAGTTGCTGTGATCGAGTCGAGGGGCATTCTCAAAGCTCATTGTTGGGGCCTGAGTTCGTTGAGTATGATGAGCCTCTCCCTTTCTCCAGCCATGAATTAATCTCCATTGCTACGGATTTGAACAAGATTGCATGGATTAAGAGTGGCCTTGAGAGTAATGGGATTAGGATGCCAGAGAACGTAGCAAGCCAGAGAGTCTTTCAAGCAGCTGCCACTACCTAG |
| *PuMYB91* | ATGAAGGAGAGACAGCGTTGGGGTGCTGAAGAGGACGCTTTGTTACGTGCATATGTGAAGCAATATGGACCAAGGGAGTGGAACCTTGTATCGCAGCGCATGAACACACCCCTAGACAGGGATGCTAAATCTTGCTTAGAAAGGTGGAAGAATTATCTCAAACCCGGCATTAAGAAAGGATCCCTAACTGAAGAGGAGCAGCGCCTTGTCATTCGTCTTCAAGCCAAACACGGTAATAAGTGGAAGAAAATTGCTGCTGAAGTCCCTGGTCGTACGGCTAAGAGATTGGGCAAGTGGTGGGAAGTGTTCAAAGAGAAGCAGCAAAGAGAACAGAAGAACAAGAAGATAACTGACCCTATTGTGGAGGGTCAATACGATAGAATACTTGAAACTTTTGCCGAGAAGTTGGTGAAGGAGCGTGCGCCAACCTATCTCATGGCTACTTCAAACGGGGCCTATCTTCATACGGAAACATCTTCTCCAGCGCCAACGATTCTTCCTCCTTGGCTTTCTAATTCCAATGTGTCCCCCAATGTGAGGCCACCATCTCCTTCTGTTACCCTGAGTCTGTCCCCGACAGTGGCACCCTCTCCGCCAATCCCTTGGCTGCAGCAGGATCGAGGATCGGATGGTAGTTTTGTTGTGGGAAATTTGCCACATCATGGTGTAGTTCCCGCTTGTGAGGAGAACCTAGTGATATCTGAGTTGGTGGAGTGCTCCAGAGAGTTGGAGGAAGTGCACCGTGCTTGGGCAGCGCATAAGAAGGAAGCTTCGTGGAGGTTAAGAAGGGTGGAGTTGCAACTGGATTCGGAGAAGGCTTGTAGGAGGACGGAGAAGATGGAAGAGATTGAAGCCAAGGTGAAGGCTCTAAGGGAAGAGCAGAAGGCTGCTCTAGACAGGATCGAAACAGAATACAGGGAACAGTTAGCAGGGCTAAGGAGAGATGCAGAAGCAAAGGAGCAGAAGTTGGCTGAGCAATGGGCCGCAAAGCATTTGCGTCTCTCCAAGTTTCTCGAGCAGATGGGAGGCAGACCAAGGATTGTCGAGCCTAATGGCCGGTGA |
| *PuMYB306* | ATGGGAAGGCCACCTTGCTGTGACAAAGTTGGTGTGAAGAAAGGGCCATGGACTCCAGAGGAAGACATCATCTTGGTCTCTTACATTCAAGAACATGGTCCTGGGAATTGGAGATCAGTTCCTACTAACACTGGTTTGCTGAGATGCAGTAAGAGCTGCAGACTTAGATGGACTAATTATCTCCGCCCGGGTATCAAACGCGGTAACTTCACTGATCATGAGGAGAAGATGATAATCCACCTCCAAGCTCTTCTGGGCAATAGATGGGCAGCCATAGCTTCATATCTTCCTCAGAGAACAGACAACGACATAAAAAATTACTGGAACACCCACTTGAAAAAGAAGCTGAAAAAGCTTCATGCAGGGCTTGATGGTCATGACCATCACAACCACCAGGATGGTTTTTCAGGTAATTCACATGATCAGCCAATCTCCAAGGGTCAGTGGGAGAGAAGGCTTCAGACTGATATCCACATGGCCAAGCAAGCTCTTTGTGAGGCTCTCTCCATTGACAAACCAAATGTTATTAGTACTACTACTGCTTCTCATGACCTTCACTTGCAAGACTTGAAGCCCTCCGTTAATATAGGTAATTACAATATTAATGATAACCCTAGCACTAGCACTAGCACTAGCACAAGGCCACACCATACCTCCACGTACGCATCCAATGCCGAAAACATTGCAAAATTGCTCGAAAACTGGATGAAAAACTCGCCAAAGGTTTCCGTTCATCAAACAAACTCGGATCAGACCAATGCTGATCAGACCAATTCCTTCAACAACAACAACAACGACAACGACAACAAAGGTGGCGCTACTGCAACTACTATAGGTCGTTCAATGAGCACTTCTAGTGAAGGGGCGCACAGCGCAACCACCACGGCGGATCAGGCTTTCGATTCGTTGTTCAGCTTCAACTCATCCCCATCGGATGTGTCTCAGTCCATGTCAGTGGATGAGAACAATGCAAATTTCATAGCAGATCAGGCAAGCTGTCTGTTTCAGGATGAGAGCAAGCCCAATTTGGAGGGACAAGTCCCTCTCTCATTGCTGGAGAAGTGGCTCCTTGACGATACTGCACCTCATGCCCACGAAGACCTAATCAACATATCATTAGAGAATGGTGCAGGCTTGTTTTGA |
| *PuMYB1R1* | ATGTCGTCCGGCACGTGCTCCACCGTCGAGCCCGCGGGCGCCGGAGAATTCATGCTGTTCGGCGTGCGCGTGGTGGTCGATTCCATGAGGAAGAGCGTCAGTTTGAACAATCTCTCGCAGTACGAGCAGCCTCAGGAGGCCGCCTCCAACAACGGCAATAACGGCACCGCCGCCGGAAAGGATGACGCGGCGCCCGGTTACACTTCCGAGAACGACGTCGTCCACAATTCTGGCGCGAATCGCGAGCGCGAACGCAAGCGAGGGGTTCCATGGACGGAGGAAGAGCACAAGCTTTTCTTGTTTGGATTGCAGAAAGTAGGGAAAGGAGATTGGAGAGGGATCTCAAGAAACTTCGTGAAGACTCGCACCCCGACTCAGGTTGCCAGCCACGCACAGAAGTACTTTCTGCGCCGGAACAACCACAATCGCCGGCGCCGCAGATCTAGCCTCTTTGACATCACCACCGATACGGTCTCTCCAACTCCAATGGATGAAGAACAAGTACATCTTCAAGATATCGCATCTCAGTCGCATCCATTGCCTCCTCCGCCACTATCCGACCCTCGCAACGCCAGTGGATTTCCAATGGTGCCAAATTTTCCAATGACTGTAGGTCCAGCTGTCTTGCCAGTTCCCATTGAGAATCCTACGGAAAATCTAGCTCTACGACAAGCAAATCACGAGAATAGTGCTTCGGCTAAGCTCGTCCGTCCAATTGCCCTTCATTCGGCCCCTCATGCCACCGCAATGTCTGATCTAAACTCGACCTCAACAATGGACACATCAACTCTCACTCTCAACCTCTCCTTGTCAATGGACTCAAGGGAACCGTCATCAACGCATTCGGCTTTCCAGACAATGTCCGGATTCAGCAATGGGGATGGCGTGATCAGCGTCGCTTAA |
| *PubHLH13* | ATGAAGATTGAGATGGGTTTAGGAGGAGGGGTTTGGGATGATGAAGATAAAGCCATGGTTGCTCAAGTTTTAGGAACCAGAGCTTTCGATTACTTGATTTCGAGCGCGGTTTCCTCGAATGAGAATTCATTTATGGGCATAGGGACTGATGAGAATTTGCACAACAAGCTCTCGGATCTCGTGGAGCGTCCGAATGCATCGAATTTTAGCTGGAATTATGCCATTTTCTGGCAGATTTCTAGGTCCAAGTCTGGGGATTGGGTTCTGTGTTGGGGTGATGGTTCTTGTAGAGAACCTAAGGAAGGTGAAGTGTCCGAAACCACGAGGATTCTCGGTCTTAGGCTTGATGATGGGACCCAGCAGAGGATGAGGAAGATAGTGCTGCAAAAGTTGCACAATTTGTTTGGGAGCTCAGATGAGTATAATGGTGCTCTTGGATTAGACCGAGTCACCGATATGGAGATGTTCTTGCTTGCGTCCATGTATTTTTCGCTTCCAAGAGGAGAGGAGGGTCCTGGCAAGTGCTTTGCATCTGGGGAGCATGTCTGGCTCTTGGACTTGTTGAAATCAGGGTCGGAGTATTGCGTTCGATCGTTTCTTGCTAAGTCTGCTGGAATTCAGACCATTGTTTTAGTCCCAACAGATGTTGGTGTAGTTGAATTGGGTTCAGTGAGATGCATAGGGGAGCGTTTAGAGTTGTTGCAATCCATAAGGTCACTGTTCTCTACACAGTCCTCGCACATGAGGGCTAAGCCAGTGGCAGGTGTGCCGATGATTGGAAGGAGGAGAGATGAAAATGTCCACCTAACTAATCTGAGCCCTGTGGAGAGAGGGGAAGCAGTTCCCAAGATTTTCGGGCAAGATTTGAACTCAGGAAACTCAGTTCGGCCTCGTTATAGAGAAAAACTTGCTGTTAGAAAGTTGGAGGAGAGGCCCTGGGATGTGTACTCAAATGGGAATAGGATTGCATTTTCAAGTCCTCGAAATGGTATTCATGGTTCGAATTGGCCGCACAATCATGATGTGAAACAGGGGAGCCCAACTGAGATGTATGCTTCTCAAAGTCCGGTGAATAATTTACAGGAGCTTGTCGATGGGGTCAGGGATGATTTTCGTCATAACCATTATCAACCACAGAAGCAGGTGCCGATGCAAATGGACTTTTCAGGGGCCACTTCAAGGCCTTCCGTGGCTCCCCGGCCCATTGGTGTGGATTCTGAAAATTCAGACGCTGAAGCTCCATGCAAGGAAGACCAGCCAGGCACAGCTGATGAAAGGAGGCCACGGAAAAGGGGTAGGAAGCCTGCAAATGGAAGAGAAGAGCCACTCAATCATGTGGAGGCAGAGAGGCAGCGGCGGGAGAAGCTAAACCAGCGGTTTTATGCTTTAAGAGCTGTTGTGCCCAATATATCCAAGATGGACAAAGCATCCTTGTTGGGAGATGCCATTGCTTACATCAACGAGCTCCAGGCGAAGCTTAAAGTCATGGAAGCAGAGAGGGAGAACCTCGGGGGCACTTCAAGAGATGCTTCAGCCTTGGGGGCTAGCTCAGGTATGGAAATTCAGAACCAGGCACCTGAGGTTGATATTCAAGCTGTTAATGACGAGGTTATTGTAAGGGTGAGCTCCCCTTTGGATTCACACCCTGCATCAAGAGTCGTCGAAGCATTCAAAGAGGCACAAATCACGGTTGTTGAGTCAAAACTTGCAGCAGCAGACGACACTGTGCTCCATACATTTGTTGTCAAGTCTCAAGGATCGGAGCGGTTGACGAAGGAGAAGTTGATTGCAGCATTTTCCCGGGAATCCAACTCTTTACATTCGTTGTCATCAGTCGGGTAG |
| *PubHLH15* | ATGGACTACGACTACTTACTTCCCACCACCTCTGCTCTGAACCGACCCAGAAAATCAACCATGCCGGAAGACGATATCATGGAGCTGCTATGGCAGAACGGCCAAGTCGTGATGCAGAGCCAGAACCAGAGGTCTTCAGTCAATAGTAAAAGATCGCACCAGTCCAAATACGACGTCGTATTGCCCGACGACGGGGGCGGCATCACCAGACCGACCCCCCAACCCCAACCCCAACCGCCGGCCCAGAACCCGCACCTGTTTATGCAAGACGATGAAATGCCCTCGTGGCTTCAGTACCCGCTCGTCGACGACCCCTTCTCCGCCGGCCTCCTCTATCCCGATTCCAGCACCTCCGAGCACCGGACGCCCCAGGTTTCGGGGCCGGCGCCAGCTTCGAGGCCGCCGATCCACCGGCCGAGGAGGACCGAGCTCCAGAACTTCCTGCAGTCCGACAGGACCAACAACAACAGGCCTATGATGTCGGAAATGGCGCCGTCGAGCTCGAAGAAGAGCGTGGTAAGGGAGACGACGACGGTCGTGGATTCCAGCGACACTCCGCTGGTGGGCCCAAGCTCTAGGGCTTTGGATTCTAGGCCCGACGGCGCTGGTGGCGGCTTGGCTAATGGAGCGAGGTCGTTAACCGCTGCAACCGCTGCGACGTCGTTTCCTGGAAAAGAAGTGACCACGTGTGAGATGTCGCTGACGTCGTCTCCAGGAGGCTCCAGCGCGAGCGCTAGTGAGAGCACCGAGCCGGAGTCGGCTCCCAAGCCGCCGCTGACTGCCGATAACCGGAAGCGCCAGCGGAAAGGGAGAGAAGCCCCCGCCGCCGACGACACCGAGTTTCAAAGCAAGGATGTCGAGTTTGAATCTGGCAATGGAAAGAAACAAGTCCGAGGATCGACATCATCTACAAAGAAATCGCGAGCTGCAGAGGTCCACAATCTCTCCGAGAGGAGACGTCGAGATAGAATAAATGAAAAGATGAAGGCTTTACAAGAACTAATACCTCGATGCAACAAGTCGGACAAAGCATCAATGCTGGATGAAGCAATTGTGTACTTGAAATCTCTCCAGTTACAAGTACAGATGATGTCCATGGGATACGGCATGATCCCTATGATGTTTCCTGGAGTTCAGCAGATGATGTCCGTGCCCATGGGGATGGGAATTGGAATGGGCATGGGCATGGGAATGGAAATGGCCGGCATCAGTCGCCCAATGATGCCATTTCCAAATGTAATCGCCGGTTCACCCATGCCAACAGCAGCTGCTGCACATATGGGACCTAGGTTCCCTATACCACCATTTCACATGCAGCCTATTCCTGCAAGTGATCCTACCGGAGTTCCAGCAGTCAACCAGACAGATCAAATGATAAACTCGCTCGGGGCACAAAATCCAAACCAATCACACATGCCAAATTTTGCAGATCCTTACCAGCAGTTTTTCAGTCCCCAACAGATGCAGTTGCCACTGCAGCACAAGCAAGCAATGCCCCAGCCAACTACTGGCAAGCCGAGTTCCAATAGGAGACCTGAAACTCATGAAAACCATCAATCAGGTTAA |
| *PubHLH21* | ATGAGAACCACCGCAAAAGGCAACCAGGAGGAGGACGAGTACGACGACGAGGAGTTTGGCTCCCGGAAAGAGGGCCCTTCTTCCAATTCCAACAGCAAGGATGCCAAGAACAATGATAAGGCTAGTGTTATACGATCGAAACATTCCGTCACGGAGCAGAGGAGGAGGAGCAAGATCAATGAGAGATTCCAGATATTGAGAGATCTAATACCCCATAGTGATCAGAAAAGAGATACAGCATCATTCCTGCTAGAGGTGATCGAGTATGTCCAGTACTTACAGGAAAAAGTACAAAAATATGAGGGTTCATACCAGGGTTGGAGTCCTGAGCCGACAAAATTAATGCCATGGAGAAATAGTCACTGGCGTGTTCAGAGTTTTGTTGGAAATCCTCAGGCCATAAAGAGCGACGCTGCACCAGTGTCACCATTTCCTGGAAAATTTGATGACAATAACATCTCTATCAATCCGAACATGCTGGCAAACAGCACACCGAATCCAGTAGAATCTGACCCTAGGAGGGATGTTGCCTCCAAAATAGTGGATCGGCAACCTGAGATAGTGAACAAGGGGATTCCTCTGCCTATGCCTCTACCAAACATGACTCCCCCGGTCAGAAGTGATGGTATGCTTGCCCAACCTCTCCAGGGACAAATTTCTGATGCTCAATCAACTCAGTGCCCCACCACTAGCGCTAGCGATGTACAGAGCCAACAGGAGGAGCTGACAATTGAAGGTGGAACAATAAGCATCTCAAGCGTTTACTCACAAGGGTTATTAAACTCTCTGAGCCAAGCGCTACAGAGTGCAGGTGTTGATCTGTCGCAGGCTAGTATCTCAGTGCAGATTGATCTTGGGAATCGTGCAAACCGAGGGCTGTCCTCTGGAACACCTGCCTCTAAGGATAATGATAATCCCCACTCAAGCAATCAGACGATGGCGCATTTTAGGGATGCTGGCAGCGGAGAGGACTCCGATCATGCCCATAAGCGCCTCAAGACATAG |
| *PubHLH66* | ATGCAGCCTTGTAGTAGAGAAATGCAGGAAATGAACTCCTTGTTGAACTCCTCAGCCTCCTCTTCCCAACTCTCCCTCCAAGACCACCACCACCTCCAACAACAACCACACTCTCACTCCCAGATGCATCATCAAATTCCAACCCCCTCCGCATCCCACTTCGACTCCGCCACCCACGATGACTTCCTCGAGCAAATGCTCTCCACCCTCGGCCCCTCCTGGGCCTCTGCCGCCGACGATGCCCCGCCTCCGCTCTCCTCCAACCCCGACAATGTCGTCTTCTCCTACGACGACTCCGCCACCCTCGCCGCCAAGTTTCGCAGCCAGCAGATCAGCGCCGGTTCCGGCGCTAACAAGTCCGCCTCCGCATCAGCCGCAGCTGCTGCCGCCATGATGCTCCAGCACCAGCTCATGATGTCCAGATCAGGCGCCGCCGACTCCGGCTTTGGTCCCACGGGATTGTCTTTGGGAACCAACGGCGACTTTGACCGCTCCAACAACGACGTCGGCGACGGCTCCTCCTTCAAATCCCCCAATCAAGTGGGCGGCGATGGGGCTTCTCCTGTTCAATCCCTGTTCAATGGCTTCGGTGCATCACTGCATGGAGCTGCTAATTCTCAGTCTCCAAATTTTCACCACCCTCAGGGAGGTCAGCTGCAAGCGCAGAACTACGGAAGTGCAGGGCCGGCGGTGAACCAAGCTCCGGTGGGTGGCTCGGCTGGGGCAGCGCCGGCCCAACCCAGGCCCAAAGTCAGGGCGAGAAGAGGTCAAGCCACTGACCCACACAGCATAGCCGAAAGATTACGGAGAGAGAGAATTGCAGAGAGAATGAAGGCTCTGCAGGAGCTCGTTCCCAATGCCAACAAGACAGACAAAGCTTCAATGCTGGATGAGATAATCGATTATGTAAAATTCCTCCAGCTCCAAGTCAAGGTTCTGAGCATGAGCAGGTTGGGCGGTGCAGGTGCTGTTGCTCCCCTTGTTGCTGATATGTCCTCCGAGGGTGGCGGTGATTGTATCCAAGCCAGTGCCAACGGCGGGACCCGCGGACGGAGCTCAAACGGCAACCAAACGGCCTCCTCATCTAACGACAACAGCATGACGGTGACGGAACACCAAGTCGCCAAGCTCATGAAGGAAGACATGGGCTCCGCCATGCAGTACCTCCAGGGAAAAGGCCTCTGCCTCATGCCGATTTCGTTGGCCACCGCCATCTCCAGCGCCACATGTCACTCCAGGAACCCCCTCCTCCACAACAACAGCAACAACCACCAAGTTGTCCCGTCCAACGGTGGCGACGGGCCCTCCTCTCCCAGCATGTCCGTGCTGACCGTCCAGTCAGCCACCATGGGTAACGGTGGGGTTGACGGTTCCGTCAAGGACGCCACCTCCGTCTCCAAGCCCTGA |
| *PuWRKY20* | ATGGACTCGATCGCACTCGACCACCCATCCGGACCCTCCGATGACGTCGATCCGGGTCTAACCGATTTCCAGCCCAGCTCCGAGCCGGCCTTTTACTCTTCTGCTGCTGCTTCCGGTGGGGCCAAGTACAAGCTCATGTCCCCGGCCAAGCTTCCGATCTCGAGGTCCCCCTGCCTCACTATCCCTCCCGGCCTCAGTCCGACGTCGTTTCTCGAGTCCCCCGTCCTCCTCTCCAACATGAAGGCAGAACCTTCCCCAACTACTGGGTCCTTTTTGAAGCCTCAAATGTTGTACGGATCTCTGAGTTCTACTACATACTCAGCAACCACAGTTTGCTCAGATTTCAATACCTCGGATGAAAGAAATTCTGGAAGCTTCGAGTTTAAGCCCCATGCTATATCAAATATGGTTACTACAGATTATAACCATTATAGGAATGAGCAGCCTGTGCAAGTCCCAGGTCAAGCTCAGCCTCAATCACATGCATCACCAACTTTGGCTAAAAATGAGATGGCAGTCTCTTCAAATGAATTGAGTTTATCTGCACCTGTTCACACTGGAGGAGCTAGTGCACCCGTGGAAGATGATTCGGGTGACTTAACACAGAGCGAACATCCACATCCTGGGGTCCAAACATCTCAGGGTGATAACAAAGGAAATGGGCCTTCAGTAATATCCTCTGATGATGGGTATAACTGGCGAAAATATGGACAAAAGCATGTTAAAGGGGGTGAATTTCCTCGCAGTTATTACAAATGTACCCATCCTAATTGTGAAGTGAAGAAGCTATTTGAGCGATCTCATGATGGACAGATAACAGAGATTATCTACAAGGGTACACATGATCATCCTAAGCCTCAACCAAGTCGACGATATAATACTGGTGCTATGATGCCTGCTCAAGAAGAAAGATCTGACAAGGCTTCATCATTGACTGGTCAAGATGACAAACCATCCAGCATTTATGGGCAAATGTCTAGTAACAATGAACCAAATAGTACTCCTGAGCTCTCTCCTGTCACAGGCAATGATGATAGTGTAGAAGGCACAGGCTCACTGTCAAATAGGTTGCCAGATGAGGTTGATGAGGATGACCAATTCTCAAAAAGGAGGAGGATGGATGTCGGTAGCATTGATATCACACCAATTGTTAAGCCTATCCGAGAACCACGAGTTGTTGTTCAGACTCTCAGTGAGGTTGATATATTAGATGATGGATACCGCTGGCGCAAATATGGTCAGAAAGTGGTGAGGGGAAATCCTAATCCAAGGAGCTATTACAAGTGCACCAATGCTGGATGCCCTGTTAGAAAACATGTGGAGAGGGCATCGCATGATCCAAAAGCAGTTATAACTACATATGAAGGGAAACATAATCATGACGTCCCTACTGCAAAGTCTAGTAGCCATGACACTTCAGGACCAACAACTGTGAATGTCCTGTCGAGAATTAGACCAGAAGAAAGTGACACCATAAGCCTTGATCTTGGTGTTGGAATGACCTCTGGTGGCGAAATTAGAACCAACGAGCACCTGCAACTGCACTCTGATCTTGTGGAACGGCAATCTCACACGAATTCCAATTTCAGGGTTGCTCAAAATACCCCTGTTTCGACATACTATGGTGTTCTTAATGGTGGCATGAATCAGTATGGATCTAGGGAAAACCCTAGTGAATCACGCAGCATCGAAATTCGACCTTTAAATCATTCCTCATACCCATATCCACAGAACATGGGAAGAGTACTAACAGGTCCGTGA |
|  |  |
|  |  |

**Table S5 Primers used for the amplification of forward and reverse fragments of PuMYB21 and PuMYB54**

| Gene | Forward primer (5′-3′) | Reverse primer (5′-3′) |
| --- | --- | --- |
| PuMYB21-Forward | CGGGATCCAGACCTACATGCTAGATG | GCTCTAGAGAAAATTGACCTCATTGC |
| PuMYB21-Reverse | CGAGCTCAGACCTACATGCTAGATG | ATTTGCGGCCGCGAAAATTGACCTCATTGC |
| PuMYB54-Forward | CGGGATCCTGAAGACGAGAAGCTTAG | GCTCTAGAGATTTTGGTTCATTATTG |
| PuMYB54-Reverse | CGAGCTCTGAAGACGAGAAGCTTAG | ATTTGCGGCCGCGATTTTGGTTCATTATTG |

**Table S6 Interference fragments of PuMYB21 and PuMYB54 used for RNAi assay**

| Interference fragments name | sequence |
| --- | --- |
| PuMYB21 | AGACCTACATGCTAGATGGGGAAACAGGTGGTCGACAATTGCACGAAGTTTGCCTGGAAGGA  CTGACAATGAGATCAAGAACTACTGGAGGACTCATTTCAAGAAAAAGGCGAAAGCACCTTCT  GATGCCTCCGAAAGGGCCAAAACCCGCCTCTTAAGGAGACATAAACCCCATAATCAGCAGCA  ACAACAACAAAATTTGCAAGTGGATCAAGAAGTGAAAAGAATCATGTCCTTATTGGACGAAA  ATGAGACTAAACTGCCGTTATATTGGCCTCACGCAGCTGATCATCATCAGGAGCAAGGCTTCTT  CTATTCCATGCTCGATGGCAATATTGTCAATGTGTCTATGCCTGAAGCTGCCTCCAGCAATGAGG  TCAATTTTC |
| PuMYB54 | TGAAGACGAGAAGCTTAGGGAGTTGGTGGAACGATATGGACCTCATAACTGGAACGCAATAGCA  GAAAAGCTCCAAGGAAGATCAGGAAAAAGCTGTAGGTTGAGATGGTTCAATCAGTTGGACCCAA  GAATCAATAGGAACCCCTTCACAGAAGAGGAAGAAGAGCGTCTCTTAGTTTCTCACCGCATTCAT  GGAAATAGATGGGCTGTTATTGCAAGGCTTTTCCCTGGACGTACCGATAACGCAGTCAAGAACCA  TTGGCATGTCATCATGGCACGCAGATGCAGAGAAAGATCTAGGCTTAATCACTCCAAATTAATAAG  GAATACTTCTACTGCTCAAACCTCGTTCATGAGTATCATTACTAACAATAATGAACCAAAATC |

**AD-PuMYB24**

**AD-PuMYB6**

**AD-PuMYB4**

**AD-PuMYB66**

**AD-PuMYB44**


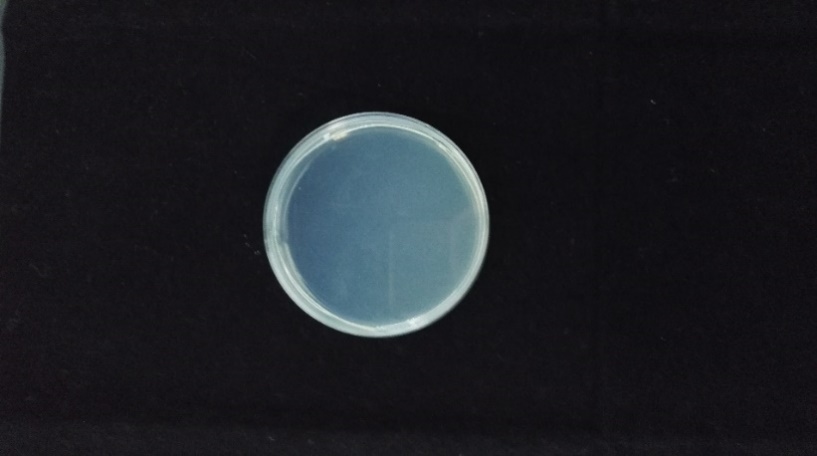

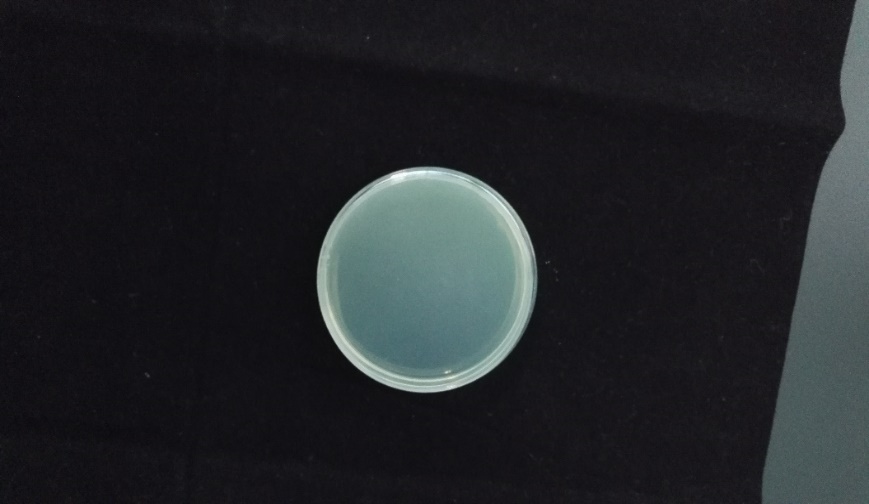

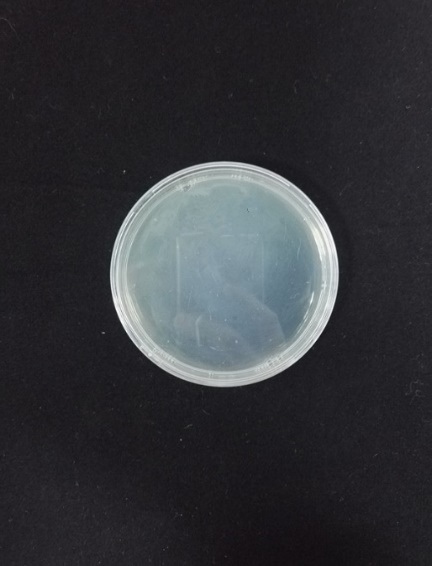

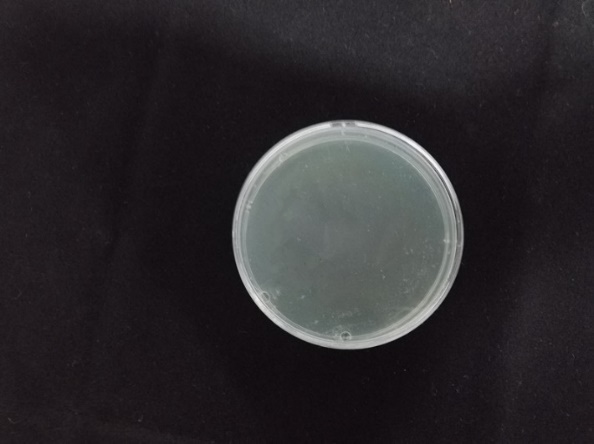

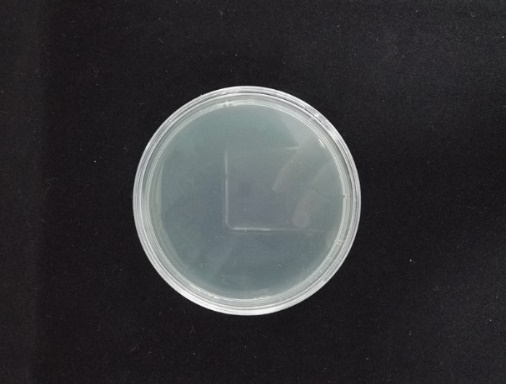


p**AbAi-*PuPLDβ1_pro_***

**AD-PuMYB1R1**

**AD-PuMYB91**

**AD-PuMYB306**

**AD-PuMYB86**

**AD-PuMYB84**


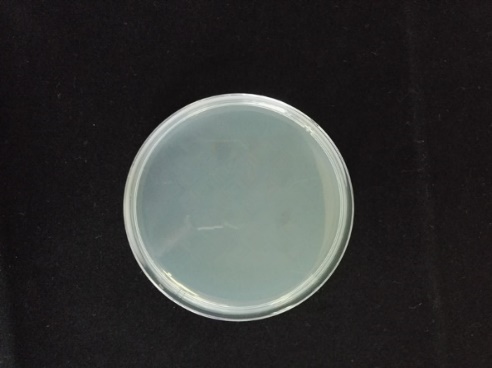

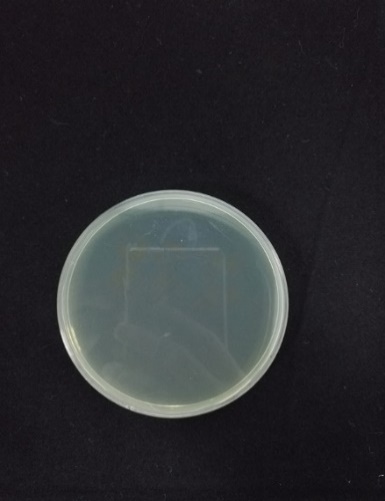

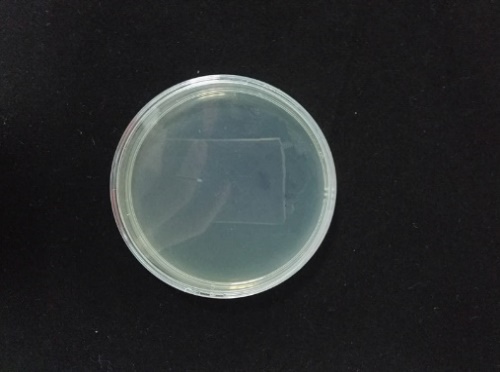

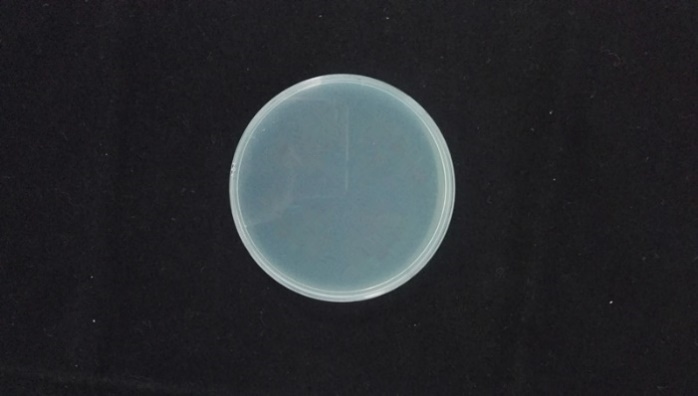

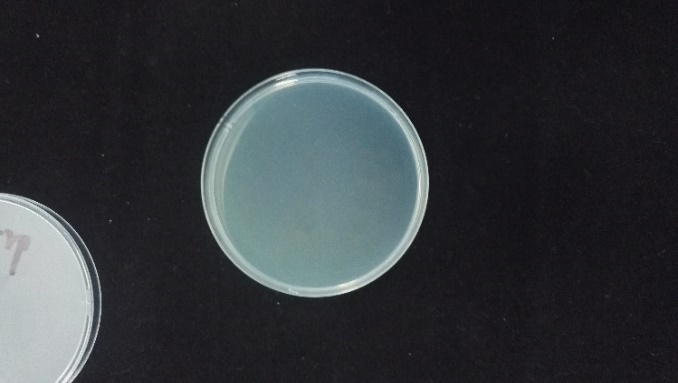


p**AbAi-*PuPLDβ1_pro_***

**AD-PuWRKY20**

**AD-PubHLH66**

**AD-PubHLH21**

**AD-PubHLH15**

**AD-PubHLH13**


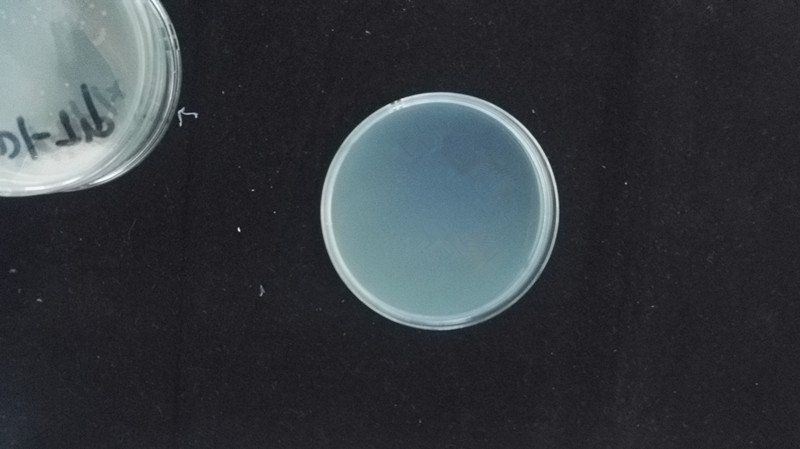

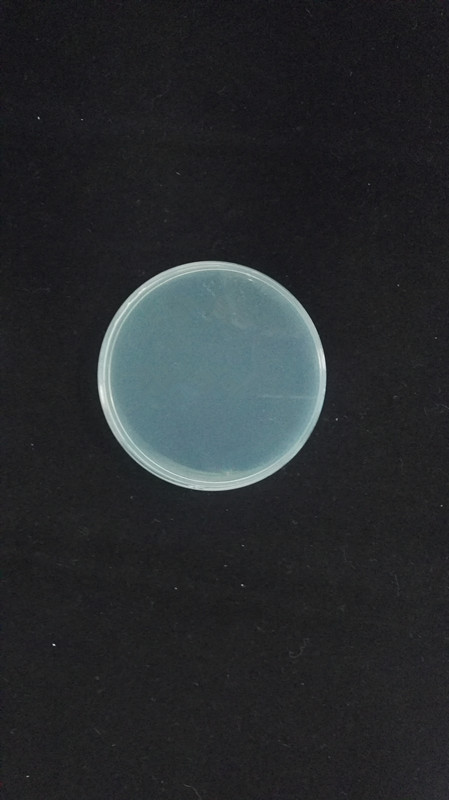

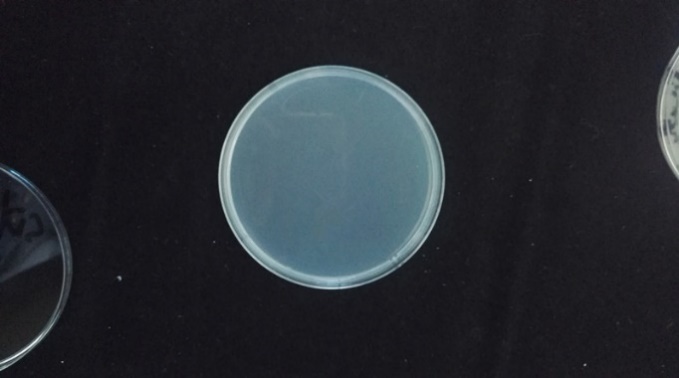

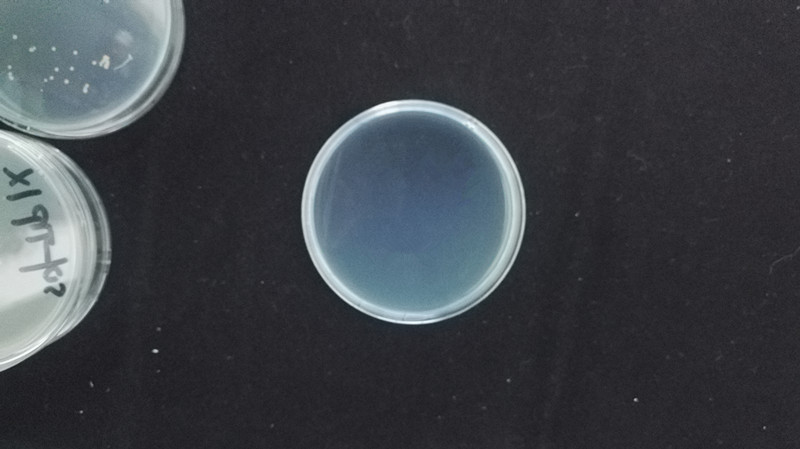

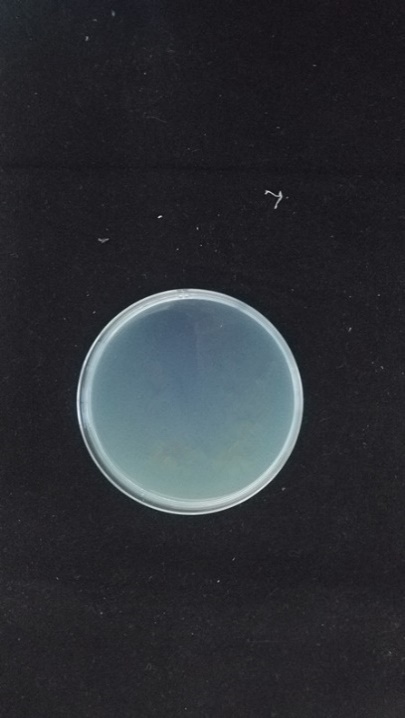


p**AbAi-*PuPLDβ1_pro_***

**Figure S1 Y1H results of other TFs on the medium containing AbA^800^**


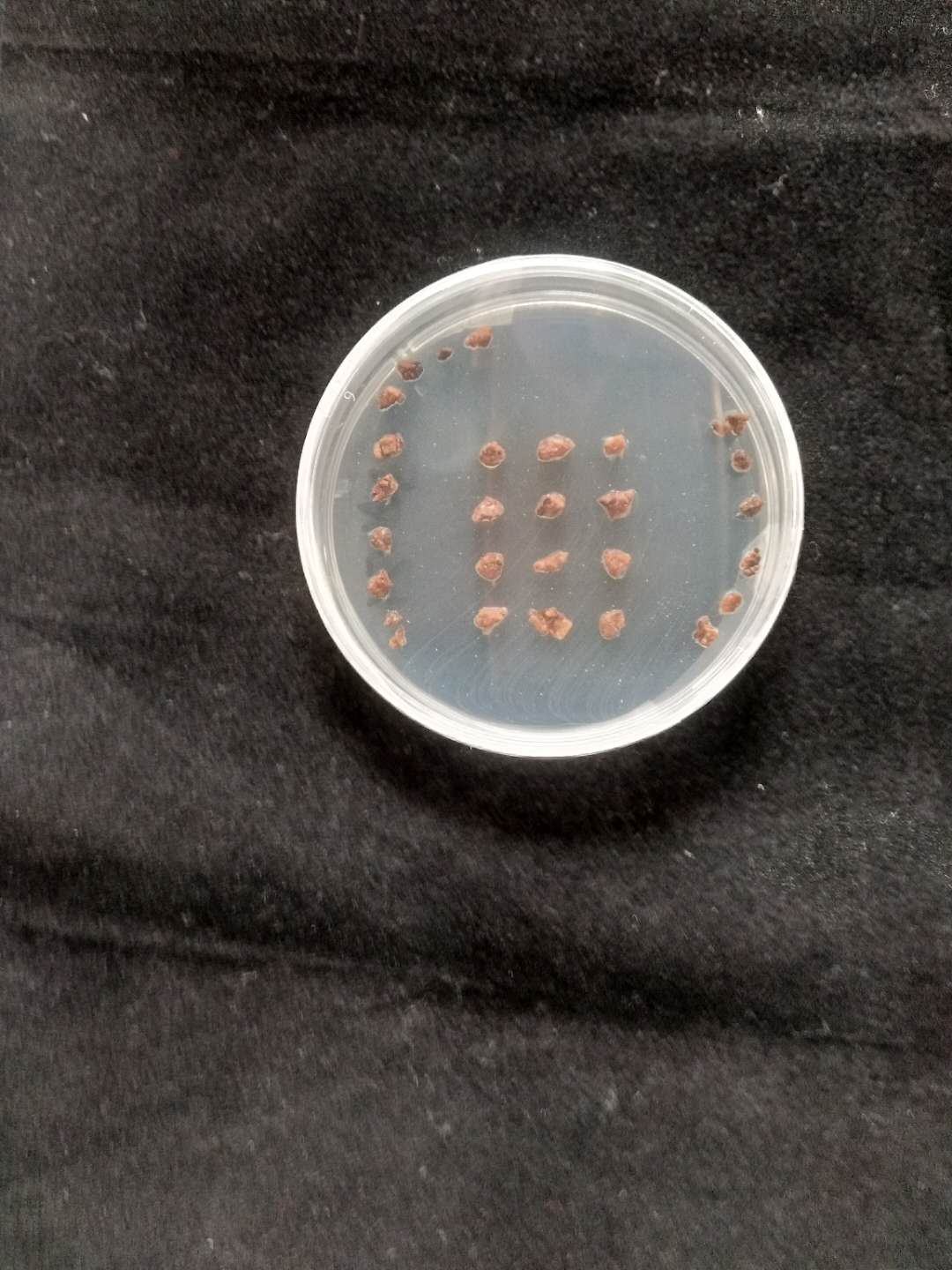

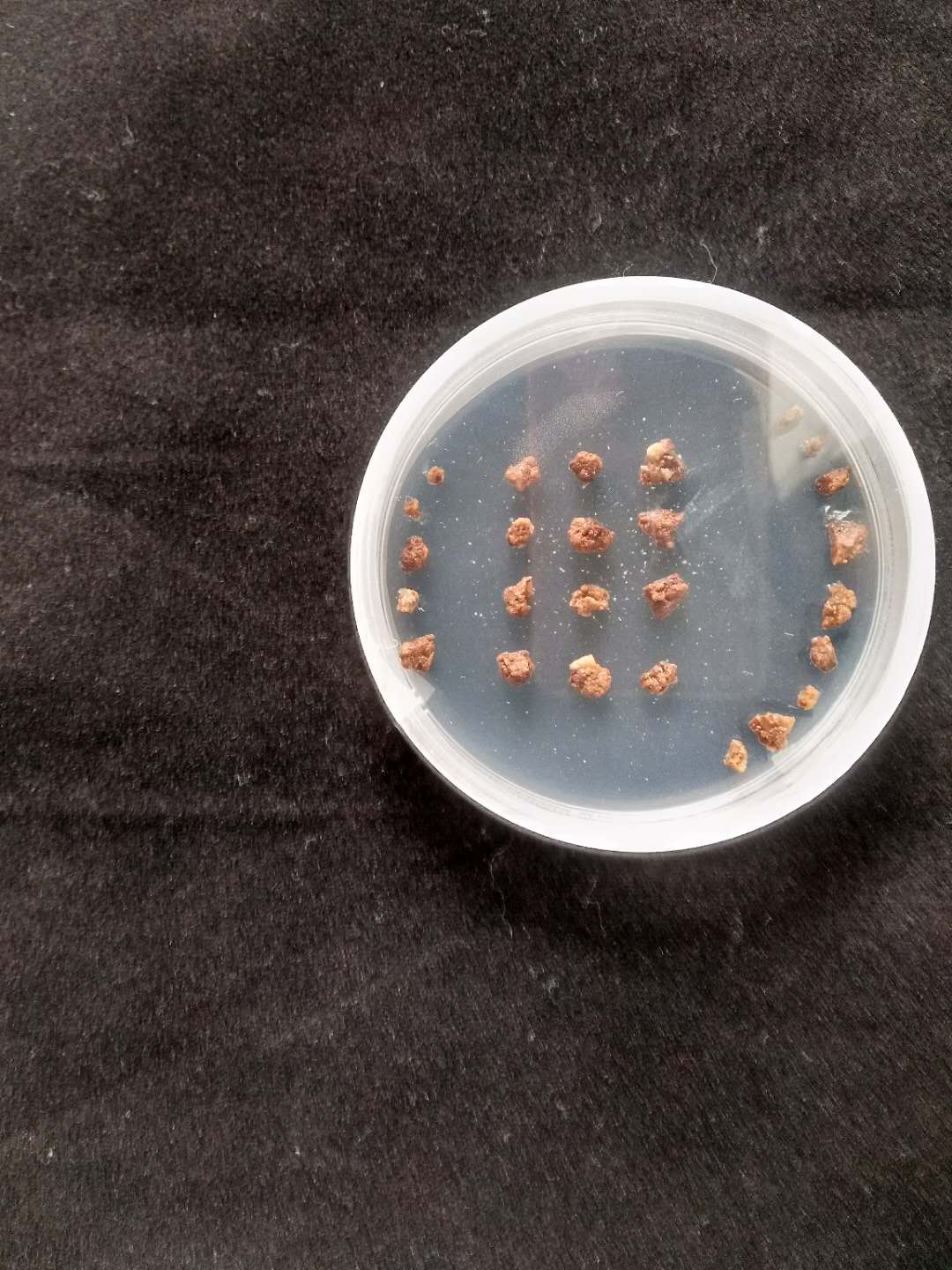


**Figure S2 ‘Nanguo’ pears calli used for transient transformation**
